# Development of Customized [^18^F]Fluoride Elution Techniques for the Enhancement of Copper-Mediated Late-Stage Radiofluorination

**DOI:** 10.1038/s41598-017-00110-1

**Published:** 2017-03-22

**Authors:** Andrew V. Mossine, Allen F. Brooks, Naoko Ichiishi, Katarina J. Makaravage, Melanie S. Sanford, Peter J. H. Scott

**Affiliations:** 1 Department of Radiology, University of Michigan Medical School, 1301 Catherine St., Ann Arbor, MI 48109 USA; 2 Department of Chemistry, University of Michigan, 930 North University Avenue, Ann Arbor, MI 48109 USA; 3 Interdepartmental Program in Medicinal Chemistry, University of Michigan, 428 Church St., Ann Arbor, MI 48109 USA

## Abstract

In a relatively short period of time, transition metal-mediated radiofluorination reactions have changed the PET radiochemistry landscape. These reactions have enabled the radiofluorination of a wide range of substrates, facilitating access to radiopharmaceuticals that were challenging to synthesize using traditional fluorine-18 radiochemistry. However, the process of adapting these new reactions for automated radiopharmaceutical production has revealed limitations in fitting them into the confines of traditional radiochemistry systems. In particular, the presence of bases (e.g. K_2_CO_3_) and/or phase transfer catalysts (PTC) (e.g. kryptofix 2.2.2) associated with fluorine-18 preparation has been found to be detrimental to reaction yields. We hypothesized that these limitations could be addressed through the development of alternate techniques for preparing [^18^F]fluoride. This approach also opens the possibility that an eluent can be individually tailored to meet the specific needs of a metal-catalyzed reaction of interest. In this communication, we demonstrate that various solutions of copper salts, bases, and ancillary ligands can be utilized to elute [^18^F]fluoride from ion exchange cartridges. The new procedures are effective for fluorine-18 radiochemistry and, as proof of concept, have been used to optimize an otherwise base-sensitive copper-mediated radiofluorination reaction.

## Introduction

Positron emission tomography (PET) imaging is a functional nuclear medicine imaging technique in which radiotracers (bioactive molecules labeled with a positron-emitting radionuclide) are administered to a patient or animal^1, 2^. When a positron is emitted from the radiotracer, it annihilates with an electron and generates two 511 keV gamma photons that are detected by the PET scanner. Mapping these gamma rays over the entire duration of the PET scan leads to an image with high spatial resolution that can provide functional information about biochemical and metabolic processes in the body. Fluorine-18 is the most common PET radionuclide because of its excellent imaging properties (a clean decay process involving 97% β^+^ emission and limited positron migration, leading to highly resolved images), favorable half-life (109.77 min), and the ready accessibility of Curie amounts of no-carrier-added [^18^F]fluoride from small medical cyclotrons.

**Figure 1:**
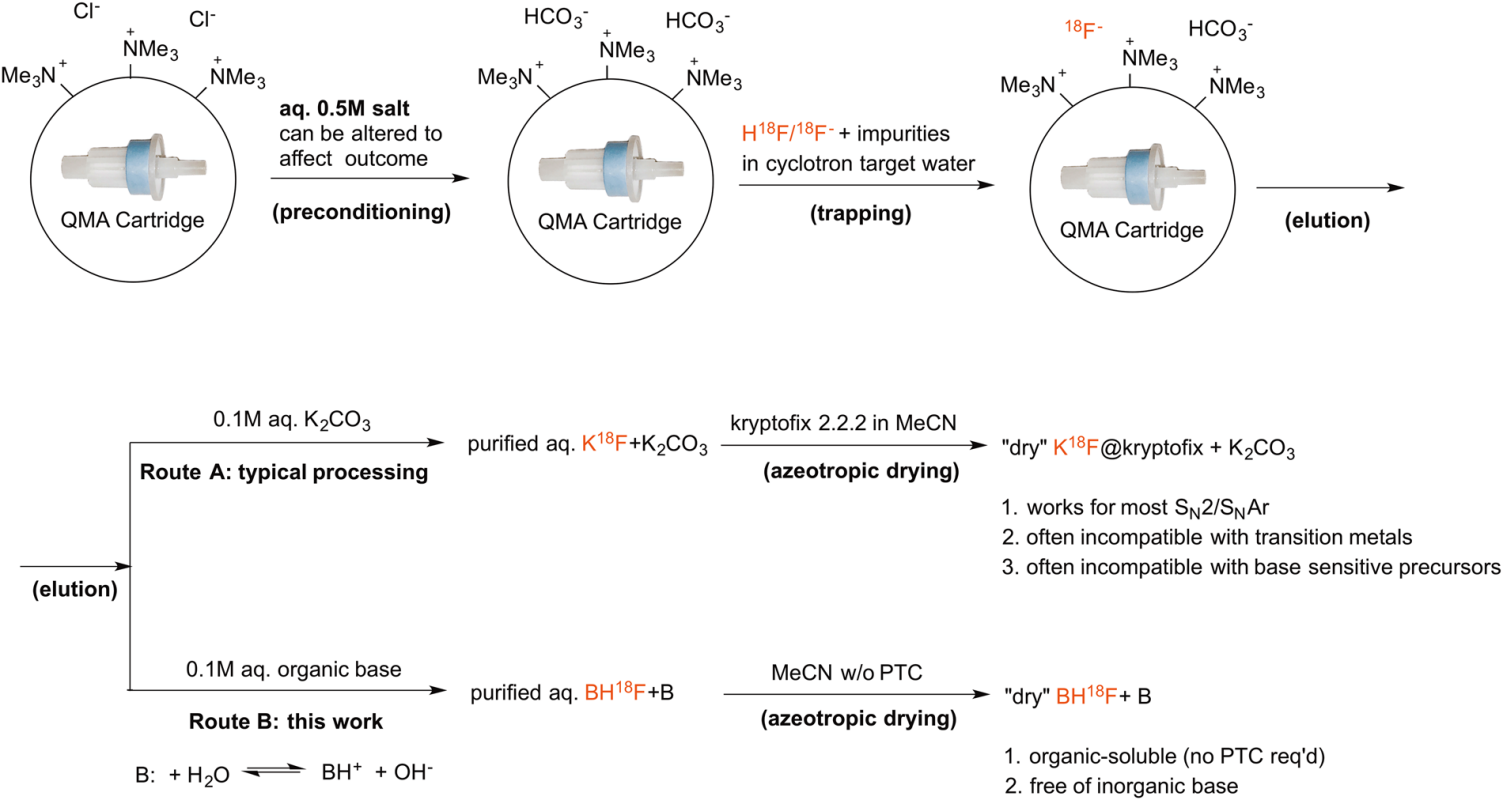
Typical fluorine-18 processing using (**A**) conventional approach and (**B**) the new approach described in this manuscript (B = Base).

[^18^F]Fluoride is produced in a cyclotron by bombarding a target loaded with [^18^O]H_2_O, via the ^18^O(p,n)^18^F nuclear reaction, and then delivered to an automated radiochemistry synthesis module as a solution in [^18^O]H_2_O. [^18^F]Fluoride is generally considered to be strongly hydrated in polar protic solvents and thereby deactivated for nucleophilic reactions (although several recent examples suggest that this might not always be the case)^3–6^. As such, a well-established three-step process is typically used to increase the reactivity of nucleophilic [^18^F]fluoride (Fig. 1)^7, 8^. This involves: (i) trapping [^18^F]fluoride on an ion exchange cartridge to recover [^18^O]H_2_O and remove impurities; (ii) eluting of X^+18^F^−^ (X = K^+^, Cs^+^, or R_4_N^+^) into a reactor using aqueous base (e.g. K_2_CO_3_, Cs_2_CO_3_ or R_4_NHCO_3_) followed by the addition of acetonitrile (and a PTC/metal-chelating crytpand such as kryptofix 2.2.2 (K_2.2.2_) if needed), and (iii) azeotropic drying of the resulting [^18^F]fluoride salt. After drying, the “activated” [^18^F]fluoride is employed in nucleophilic radiofluorination reactions. This classical approach has worked satisfactorily for numerous nucleophilic radiofluorination reactions^9^ since its introduction in 1986^10–13^.

Recently there has been renewed interest in developing novel approaches for “late-stage fluorination”^14–19^. Spectacular advances in transition-metal catalyzed fluorination reactions, as well as the development of many new precursors amenable to late-stage fluorination, have greatly expanded the scope of radiopharmaceuticals that can be accessed from [^18^F]fluoride^14, 17^. However, the complicated reaction pathways involved in these new reactions have exposed limitations in the traditional method for handling cyclotron-produced [^18^F]fluoride. For example, the Cu-mediated fluorination of organoboron compounds, originally developed by the Sanford group^20^ and subsequently adapted for radiolabeling by Sanford and Scott^21^, Gouverneur^22, 23^, and Neumaier^24, 25^, has been used to prepare a range of radiofluorinated arenes in small-scale manual reactions. However, automation and scale-up to the Curie levels of [^18^F]fluoride used in typical production of radiopharmaceuticals for clinical use have proven challenging^21, 24^. The inorganic bases (e.g. K_2_CO_3_) and cryptands (e.g. K_2.2.2_) used for standard [^18^F]fluoride processing result in suppressed radiochemical yields in this Cu-mediated [^18^F]fluorination reaction, possibly due to the formation of unproductive copper adducts^24^. The presence of K_2_CO_3_ and/or K_2.2.2_ can also be detrimental to subsequent reactions and/or protecting group manipulations, as we have discovered in recent PET radiotracer development efforts^26^.

To address these limitations, we considered the use of alternative eluents to process [^18^F]fluoride. Several recent reports have focused on the development of eluent systems including transition-metal complexes^27–29^, as well as bases, salts or neutral onium salts intended as milder replacements for K_2_CO_3_ and/or to reduce (or even eliminate) the need for K_2.2.2_^30–39^. For example, Lemaire and colleagues used strong bases to generate hydroxide and elute [^18^F]fluoride from ion exchange cartridges^34^, while Wessmann and co-workers showed that azeotropic drying could be eliminated if [K^+^K_2.2.2_]OH- in acetonitrile was used to elute fluoride^39^.

Building on these concepts, we sought to replace deleterious bases and/or chelating agents in [^18^F]fluoride processing with metal salts and/or ancillary ligands necessary for the metal-catalyzed reaction. We initially explored aqueous solutions of pyridinium *p*-toluenesulfonate (PPTS) or KOTf for the Cu-mediated fluorination of organoborons^21^. Based on these initial promising results, we considered whether other aqueous solutions could be used to elute [^18^F]fluoride. This would open the intriguing possibility of tailoring the [^18^F]fluoride eluent to the reaction at hand, rather than defaulting to the K_2_CO_3_/K_2.2.2_ system. Herein we demonstrate proof-of-concept by matching new [^18^F]fluoride processing conditions to the copper-mediated [^18^F]fluorination (and subsequent reactions) of aryl boron derivatives.

## Results and Discussion

### Alternative Eluent Studies

Our studies commenced with an examination of the use of copper salts from our recently reported Cu-mediated fluorination reactions to prepare [^18^F]fluoride. We initially investigated elution of [^18^F]fluoride from tetramethylammonium anion exchange cartridges (QMA cartridges) using Cu(OTf)_2_, and found that 0.025 M aqueous solutions of Cu(OTf)_2_ resulted in >85% [^18^F]fluoride recovery from the QMA (see Supporting Information (SI) for full details of Cu studies). On the basis of these initial findings, we used Cu(OTf)_2_ as the [^18^F]fluoride eluent in the automated synthesis of [^18^F]fluoroacetophenone ([^18^F]FAP, **2**) from 4-acetylphenylboronic acid (**1-B(OH)**_**2**_), using our previously reported [^18^F]fluorodeboronation chemistry^21^. However, in automated fluorination reactions, the radiochemical conversion (RCC) to [^18^F]FAP was dramatically lower when eluting with Cu(OTf)_2_ (4–5%) than with our previously reported KOTf/K_2_CO_3_ elution (yields of [^18^F]FAP were 61 ± 8% using optimized manual conditions but lower yields of 8–12% were obtained in the automated [^18^F] fluorination). Thus we next turned our attention to eluting [^18^F]fluoride using ancillary ligands that are commonly employed in transition-metal mediated reactions.

As ligands are often present in excess in metal-mediated reactions, they are generally less constrained to specific concentration ranges than the metal salt and are typically more redox stable than transition metal cations. This lessens the importance of variable final concentrations resulting from, for example, inconsistent retention on the QMA cartridge during elution. In aqueous solution weak bases undergo equilibrium protonation in water to form BH^+^-OH^−^ ion pairs. As such, we hypothesized that basic ligands or other non-ionic weak bases with a high enough conjugate pKa in water would generate sufficient OH^−^ to elute [^18^F]fluoride from QMA cartridges *via* anion exchange. We first tested a range of concentrations of aqueous KOH to determine the concentration of OH^−^ required to elute [^18^F]fluoride from QMA cartridges preconditioned with either 0.5 M NaHCO_3_ or KOTf. [^18^F]Fluoride recovery from the QMA cartridges was predicted to be related to KOH concentration (Table 1). This relationship was found to be sigmoidal in nature, consistent with previously reported ionic eluent systems^30^.

**Table 1:** Investigation of the effect of pre-conditioning agent on the elution of [^18^F]fluoride from Waters QMA-lite cartridges.

Entry	Preconditioning Agent	Fluoride recovery (%)			
		0.005 M KOH	0.0075 M KOH	0.01 M KOH	0.02 M KOH
1	NH_4_HCO_3_		15		77
2	NEt_4_HCO_3_		7		75
3	NaHCO_3_	21	34	75	84
4	Na_2_CO_3_		82		92
5	K_2_CO_3_		78		94
6	Na_2_SO_4_	62	78	78	89
7	K_2_HPO_4_		68		94
8	KH_2_PO_4_		6		67
9	KOH		68		72
10	KI		19		78
11	KBr		5		68
12	KCl		7		55
13	KOTf	0	4	29	31
14	PPTS		28		67
15	NaHCO_2_		2		29
16	KOAc		3		16

We noted that [^18^F]fluoride recovery was consistently greater from cartridges preconditioned with NaHCO_3_ when compared to KOTf, suggesting that preconditioning can also impact [^18^F]fluoride recovery. To further explore this effect, we evaluated a series of preconditioning agents, and observed a clear trend. [^18^F]Fluoride adsorption (loading) onto the QMA cartridge was not affected by choice of preconditioning agent, while [^18^F]fluoride recovery was found to be proportional to the valency and ionic character of the preconditioning salt (Table 1). These data suggest that the ability for weak bases to elute [^18^F]fluoride is dependent on both OH^−^ concentration (which can be predicted from the conjugate acid (BH^+^) pKa value), and the cartridge preconditioning agent, which can be changed to affect [^18^F]fluoride recovery and ostensibly the reaction conditions.

**Figure 2:**
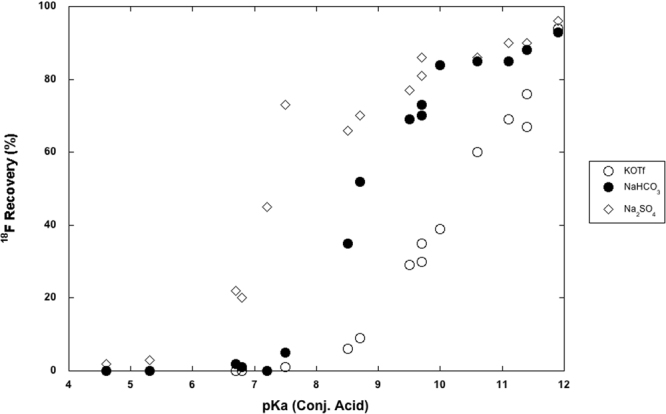
Charts of [^18^F]fluoride recovery as a function of pKa for cartridges pre-conditioned with KOTf (○), NaHCO_3_ (●) or Na_2_SO_4_ (◊).

In light of these results, aqueous solutions of weak nitrogenous bases encompassing a range of pKa values were next tested as [^18^F]fluoride eluents using QMA cartridges pre-conditioned with either NaHCO_3_, KOTf, or Na_2_SO_4_ (Table 2). As anticipated, a positive sigmoidal relationship between [^18^F]fluoride recovery and pKa of conjugate acid (BH^+^) was observed (Fig. 2). However, the weak base solutions eluted a greater proportion of [^18^F]fluoride from the QMA cartridge than would be predicted on the basis of OH^−^ concentration alone. For example, a 0.1 M aqueous solution of Et_3_N (conjugate pKa = 11.1) has a predicted OH^−^ concentration of 0.011 M, which should correspond to a [^18^F]fluoride recovery of 29–31% based on the 0.01–0.02 M KOH elution data (Table 2, KOTf preconditioning). Instead, a [^18^F]fluoride recovery of 69% was observed (KOTf preconditioning). This trend is observed for all of the bases and QMA preconditioning agents investigated, and may arise from preferential hydrogen bonding interactions between BH^+^ and [^18^F]fluoride, assisting in the desorption of [^18^F]fluoride from the QMA.

**Table 2:** Investigation of weak base solutions (500 µL of 0.1 M aqueous solution) as eluents for [^18^F]fluoride trapped on Waters QMA-lite cartridges.

Entry	0.1 M aq. base	pKa (conj)	^18^F recovery (%)		
			Cartridge precond. agent		
			KOTf	NaHCO_3_	Na_2_SO_4_
1	Aniline	4.6	0	0	2
2	Pyridine	5.3	0	0	3
3	4-OMe pyridine	6.7	0	2	22
4	2,6-lutidine	6.8	0	1	20
5	Imidazole	7.2	0	0	45
6	Collidine	7.5	1	5	73
7	Morpholine	8.5	6	35	66
8	DABCO	8.7	9	52	70
9	Ammonia	9.5	29	69	77
10	DMAP	9.7	35	73	81
11	ethanolamine	9.7	30	70	86
12	K_2.2.2_	10.0	39	84	84
13	Methylamine	10.6	60	85	86
14	trimethylamine	11.1	69	85	90
15	Diethylamine	11.4	76	88	90
16	DIPEA	11.4	67	88	90
17	DBU	11.9	94	93	96

To develop a predictive model for [^18^F]fluoride recovery vs. pKa of conjugate acids, regression analysis was conducted on the data collected in this experiment using GraphPad software (90% confidence level). Regression equations were obtained with high correlation (R^2^ > 0.9) for each preconditioning agent and serve as a crude predictor of [^18^F]fluoride recovery when similar non-ionic eluents are used (see SI for full details). For example, the equations predict that when eluting with 500 μl of a 0.1 M solution of aqueous base, then  bases with a conjugate acid pKa of ≥10.4 are optimal for recovering >50% [^18^F]fluoride from ion exchange cartridges preconditioned with KOTf, whereas pKa values ≥8.9 and ≥7.7 are required when preconditioning with NaHCO_3_ and Na_2_SO_4_, respectively.

For all weak base eluents tested, [^18^F]fluoride recovery was found to be related to preconditioning agent in the following order: KOTf < NaHCO_3_ < Na_2_SO_4_. This suggests that a combination of appropriate preconditioning and increased eluent concentration can improve [^18^F]fluoride recovery to the levels needed for radiochemical synthesis when eluting with weak bases (pKa < 7). To test this hypothesis, we chose pyridine-based eluents (pyridine and 4-methoxypyridine), as they afforded poor results with KOTf and NaHCO_3_ preconditioning, but are useful ligands for metal-mediated reactions^21^. Several divalent preconditioning agents (Table 3, entries 1–6) were re-evaluated with 0.1 M solutions of these bases, and Na_2_SO_4_ preconditioning resulted in the greatest [^18^F]fluoride recovery. Both pyridines were then examined at higher concentrations with Na_2_SO_4_ QMA preconditioning. [^18^F]Fluoride recoveries increased steadily to a maximum of 46% and 66% for 500 μL of 1 M pyridine and 4-methoxypyridine, respectively. Notably, this is equivalent to adding 500 µmol of pyridine to a reaction, which is approximately the optimal amount needed for the Cu-mediated fluorination of organoborons^21^. We also tested the impact of preconditioning agent when eluting [^18^F]fluoride with Cu(OTf)_2_, to see if this effect was unique for basic eluents, but results were less conclusive (see SI).

**Table 3:** Effect of pre-conditioning agent on the elution of [^18^F]fluoride with aqueous pyridine and 4-OMe-pyridine.

Entry	Eluent solvent	Preconditioning. agent	Total amount (µmol)	^18^F recovery (%)	
				Pyridine	4-OMe pyridine
1	Water	NaHCO_3_	50	0	0
2	Water	KOTf	50	0	0
3	Water	Na_2_CO_3_	50	0	0
4	Water	K_2_CO_3_	50	0	0
5	Water	K_2_HPO_4_	50	4	24
6	Water	Na_2_SO_4_	50	15	48
7	Water	Na_2_SO_4_	125	23	55
8	Water	Na_2_SO_4_	250	20	53
9	Water	Na_2_SO_4_	375	21	59
10	Water	Na_2_SO_4_	500	46	66

### Novel Synthesis of ^18^F-fluoroarenes Using a Novel Dimethylaminopyridine (DMAP) Elution Method

Finally, we tested whether these new elution approaches could enhance the synthesis of ^18^F-fluoroarenes that have proven difficult to access by other labeling strategies. For example, we have a long standing interest in accessing [^18^F]4-fluorophenacylbromide ([^18^F]FPB, **3**) via [^18^F]FAP (**2**), due to its potential as a PET radiotracer for glycogen synthase kinase-3 (GSK-3)^40^. Typical yields of [^18^F]FAP synthesized via traditional S_N_Ar chemistry (e.g. from the nitro-precursor) are 60–70%^41^. However, existing methods for conversion to [^18^F]FPB have proven problematic in our hands as well as when attempted by others^41^, because of the need to brominate [^18^F]FAP^42–44^. Most previously reported syntheses of [^18^F]FPB have utilized Br_2_ as a brominating reagent. However, we sought to avoid this reagent due to its volatility, toxicity, and the incompatibility of our synthesis module components with this strong oxidant. We likewise wished to avoid “inconvenient” reagents such as perbromide resins or other solid-phase reagents^44^, because of their incompatibility with modern automated synthesis modules. Moreover, the reported bromination methods suffer from well documented reproducibility issues^41^. We hypothesize that these reproducibility problems stem from complication of the acid-catalyzed bromination by the presence of residual K_2_CO_3_ and/or K_2.2.2_ from [^18^F]fluoride processing. As such, this is an ideal system for proof-of-concept demonstration that elution conditions can be customized to enhance a given radiochemical synthesis.

**Figure 3:**
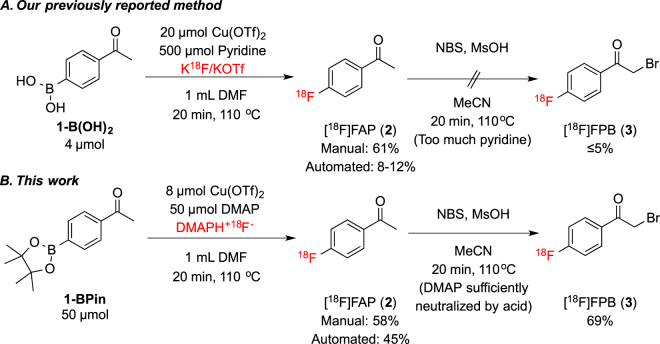
Comparison of existing method for the [^18^F]fluorination of arylboronates (**A**)^21^, and new modified method (**B**). Changing the base from pyridine to DMAP is necessary for successful one-pot acid-catalyzed bromination of [^18^F]FAP (**2**) to [^18^F]FPB (**3**).

We previously demonstrated a Cu-mediated synthesis of [^18^F]FAP (**2**) in an automated module starting from either 4-acetylphenylboronic acid pinacol ester (4-BPin-acetophenone, **1-BPin**) or the corresponding arylboronic acid (**1-B(OH)**_**2**_), using KOTf (5 mg spiked with 50 μg of K_2_CO_3_, corresponding to a 73/1 molar ratio) to process the [^18^F]fluoride^21^. Yields of [^18^F]FAP were comparable to those obtained using traditional S_N_Ar: 61 ± 8% using optimized manual conditions and 8–12% when the process was automated^21, 41^. We used *N*-bromosuccinimide (NBS) in the presence of methanesulfonic acid for the bromination, as it is a relatively mild method for the α-bromination of ketones that is amenable to automation^45, 46^. However, attempts to brominate the [^18^F]FAP (**2**) produced in this fashion proceeded in ≤5% RCC (Fig. 3a), and provided insufficient quantities [^18^F]FPB (**3**) for pre-clinical imaging studies. We hypothesize that K_2_CO_3_ from [^18^F]fluoride processing, as well as pyridine left over from the [^18^F]fluorination, interfere with the acid-catalyzed bromination, in a similar manner to K_2_CO_3_/K_2.2.2_ described above.

In our studies of the radiofluorination of arylboron reagents, we discovered that DMAP can be used in place of pyridine for the [^18^F]fluorination of certain organoboron derivatives. While DMAP is not compatible with the fluorination of boronic acid precursors, likely due to the presence of acidic protons, we obtained modest RCC values (20–40%) when fluorinating arylBpin derivatives using a 1: 2: 2.5 ratio of arylBpin: Cu(OTf)_2_: DMAP^21^. As aqueous solutions of DMAP can be used to elute [^18^F]fluoride at similarly low concentrations (Table 2), this serves as an ideal system to test this new elution strategy.

We first optimized the radiolabeling reagent loading to accommodate the amount of DMAP (500 µL of a 0.1 M solution, or 50 µmol) required for satisfactory [^18^F]fluoride recovery. In preliminary studies, the highest RCC was observed with NaHCO_3_ preconditioning (5%). Given the potential improvements offered by QMA preconditioning with Na_2_SO_4_, we also tested this possibility. However there was no product formation, likely due to deactivation of the catalyst via the coordination of SO_4_^2−^ to Cu^2+^. This result was not entirely unexpected, as it is in line with our previous findings that CuSO_4_ is an inadequate catalyst for this chemistry^21^ and prior reports have shown that QMA preconditioning agents end up in reaction mixtures^32^. This finding demonstrates the need to carefully consider each aspect of fluoride processing when designing and/or optimizing late-stage fluorination approaches.

Through a further series of reaction screens (see SI), we found that 50 µmol of 4-BPin-acetophenone (**1-BPin**), 8 µmol of Cu(OTf)_2_, and 50 µmol of DMAP in 1 mL of DMF led to an optimal 58% RCC to [^18^F]FAP (**2**) when the reaction mixture was heated to 110 °C for 20 minutes (Fig. 3b). Importantly, the 50 µmol DMAP used in this process is an order of magnitude less than the 500 µmol of pyridine required in our original method^21^, and was therefore expected to be far less of a complicating factor in the subsequent bromination step.

We next revisited the automated synthesis of [^18^F]FAP (**2**) and subsequent conversion to [^18^F]FPB (**3**). The optimized method, using NaHCO_3_ as the preconditioning agent, was transferred to a TRACERLab FX_FN_ synthesis module. The order of reagent addition was then evaluated to examine if it impacts the reaction (see SI for full details). The best yields were obtained by pre-mixing a solution of [^18^F]fluoride and arylBpin **1-BPin** at 100 °C for 5 min, followed by addition of Cu(OTf)_2_ and DMAP. Conducting the reaction at 110 °C for 20 min resulted in 45% RCC to **2**. This result suggests that dissolution of [^18^F]fluoride is a critical step in this synthesis, and must occur prior to the addition of [^18^F]fluoride or substrate to Cu(OTf)_2_. Mechanistic studies into these effects are currently underway.

Finally, investigation of the bromination of [^18^F]FAP (**2**) to generate [^18^F]FPB (**3**) revealed that subjecting [^18^F]FAP, prepared from **1-BPin** using the new DMAP elution method, to identical bromination conditions as those described above (NBS/MsOH) resulted in 69% RCC to [^18^F]FPB (**3**) (the dibrominated product was also obtained in 24% RCC). Putting this all together and running a fully automated synthesis of [^18^F]FPB provided 13 mCi of isolated and formulated product (1.5% non-corrected radiochemical yield (RCY), 99% radiochemical purity and 8,097 Ci/mmol specific activity), enough to conduct GSK-3 preclinical PET imaging in rodents and primates and these studies are ongoing.

### Substrate Scope of DMAP Elution Method

**Figure 4:**
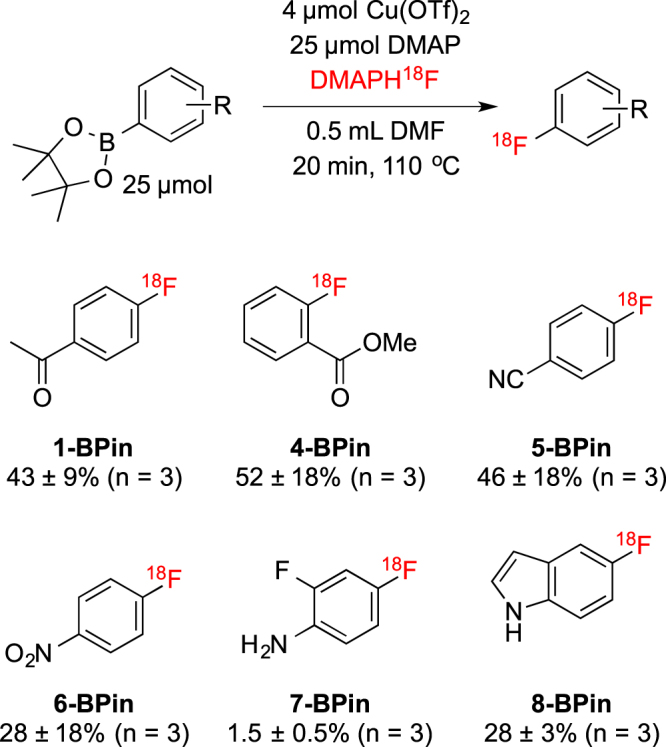
Substrate Scope RCCs. Conditions: 25 µmol of BPin, 4 µmol of Cu(OTf)_2_, and 25 µmol of DMAP in 0.5 mL of DMF, DMAPH^18^F, 110 °C, 20 min.

While these reaction conditions were specifically designed to enable an automated one-pot two step synthesis of [^18^F]FPB, we sought to establish whether they were generally applicable to the fluorination of arylBPin esters. In addition to **1-BPin**, we subjected a small series of substrates (**4-BPin** – **8-BPin**) to the optimized conditions, and found that the method was suitable for fluorinating a range of different arylBPin esters (Fig. 4). In general, yields were comparable to those obtained using our previously reported radiofluorination of arylboron reagents^21^. Both methods are tolerant of a variety of functional groups and unprotected heteroatoms and, like our recently developed radiofluorination of arylstannanes^47^, can be conducted under an inert atmosphere making then straightforward to automate using radiochemistry synthesis modules. The results suggest that for many substrates these methods are interchangeable. However, for complex precursors, or cases where further chemistry is required downstream on labeled intermediates, one or the other can be selected (and/or further optimized) using the strategies introduced herein.

### Conclusion

In conclusion, this paper introduces the concept of tailoring [^18^F]fluoride processing conditions to enhance late-stage radiofluorination reactions. The appropriate choice of [^18^F]fluoride processing conditions can lead to milder, simpler, and higher yielding radiofluorinations as well as improve downstream reactions with labeled intermediates. For example, we have demonstrated that aqueous solutions of non-ionic bases and/or ligands used in copper-mediated fluorination reactions, in combination with an appropriate QMA preconditioning agent, can be utilized as eluents for fluorine-18 processing. The resultant conjugate acid [^18^F]fluoride salts are fully soluble in most organic solvents, negating the need for additional phase transfer catalysts. As a proof-of-concept, we show that an elution method using DMAP enables the copper-mediated synthesis of [^18^F]FAP and subsequent conversion to [^18^F]FPB, which has proven problematic using other synthetic approaches. Ultimately, we anticipate that the concept of tailoring [^18^F]fluoride processing conditions to a given reaction will prove broadly applicable to diverse radiofluorinations in the future.

## Methods

Full details of experimental procedures as well as associated analytical data can be found in the Supporting Information.

### General [^18^F]fluoride elution studies method

Waters QMA-light Sep-Paks were washed sequentially with ethanol (10 mL), 0.5 M preconditioning agent in water (10 mL), and deionized water (10 mL). Aqueous [^18^F]fluoride (0.5 mL) was passed through a QMA cartridge followed by 2 mL air, and the activity of the QMA cartridge was determined with a Capintec dose calibrator. [^18^F]Fluoride was then eluted from the QMA cartridge into a 4 mL vial with 0.5 mL eluent solution, followed by 2 mL of air. Activity of the 4 mL vial (eluate) and QMA cartridge (residual [^18^F]fluoride) were determined with a Capintec dose calibrator. Activity data was used to calculate % fluoride recovery.

### General radiofluorination details

Fluorine-18 was produced via the ^18^O(p,n)^18^F nuclear reaction using a GE PETTrace cyclotron (40 μA beam for 2 min generated ca. 150 mCi of fluorine-18 as measured by synthesis module detector). The [^18^F]fluoride was then processed and either employed in manual reactions, or automated syntheses using a TRACERLab FX_FN_ radiochemistry synthesis module, according to methods described in the SI. Total recovered activity at end-of-synthesis was measured with a Capintec dose calibrator.

### Quality control analysis

Reactions were analyzed by radio-TLC using a Bioscan AR-2000 TLC scanner, and/or HPLC using a Shimadzu LC-2010A HT system equipped with a Bioscan B-FC-1000 radiation detector, according to the methods described in the SI.

## Electronic supplementary material

Supporting Information
