# Supplementary material for: Development of Customized [18F]Fluoride Elution Techniques for the Enhancement of Copper-Mediated Late-Stage Radiofluorination
Source: Sci Rep. 2017 Mar 22;7:233. doi: 10.1038/s41598-017-00110-1 (PMC5427906; doi:10.1038/s41598-017-00110-1)
Supplement: Supplementary file 1 — Supporting Information [file 41598_2017_110_MOESM1_ESM.pdf]

SUPPORTING INFORMATION

Development of Customized [^{18}F] Fluoride Elution Techniques for the Enhancement of Copper-Mediated Late-Stage Radiofluorination

Andrew V. Mossine,[†] Allen F. Brooks,[†] Naoko Ichiishi,[‡] Katarina J. Makaravage,[‡] Melanie S. Sanford[*,‡] and Peter J. H. Scott[*,†,#]

[†] Department of Radiology, University of Michigan Medical School, 1301 Catherine St., Ann Arbor, MI 48109, USA

[‡] Department of Chemistry, University of Michigan, 930 North University Avenue, Ann Arbor, MI 48109, USA

[#] Interdepartmental Program in Medicinal Chemistry, University of Michigan, 428 Church St., Ann Arbor, MI 48109, USA

Table of Contents

1. Materials and Methods pS03

2. Evaluation of Eluents and Preconditioning Reagents pS03
   2.1 Preparation of aqueous fluorine-18 pS03
   2.2 [^{18}F]fluoride elution studies method pS03
   2.3 Evaluation of Eluents and Preconditioning Reagents: Raw Data pS04
      2.3.1 Copper Triflate as eluent pS04
      2.3.2 KOH as eluent pS05
      2.3.3 Preconditioning studies (KOH as eluent) pS06
      2.3.4 Preconditioning studies ($Cu(OTf)_2$ as eluent) pS07
      2.3.5 Preconditioning studies (Weak bases as eluents) pS08
      2.3.6 4-OMePyridine and Pyridine elution optimization studies pS10

3. Synthesis of [^{18}F]-4-Fluoroacetophenone *via* Manual Reactions pS11
   3.1 Preparation of [^{18}F]fluoride solution for manual syntheses pS11
   3.2 Manual synthesis method pS12
   3.3 Radiolabeling Optimization Screens pS13
      3.3.1: Reagent loading screen pS12
      3.3.2: Order of Addition screen (manual) pS15
      3.3.3: Order of Addition screen (automated) pS17
   3.4 Substrate Scope pS19

4. Synthesis in a TRACERLab FX_{FN} Synthesis Module. pS20
   4.1 General Considerations pS20
   4.2 Thin Layer Chromatography Analysis pS20
   4.3 HPLC Analysis pS21
   4.4 General Synthetic Procedures pS22
      4.4.1 Synthesis of [^{18}F]-4-fluoroacetophenone with DMAP elution pS22
      4.4.2 Synthesis of [^{18}F]-4-fluorophenacylbromide with DMAP elution pS23
      4.4.3 Synthesis of [18F]-4-Fluorophenacyl Bromide ([18F]FPB) with DMAP Elution: in-box purification and reformulation. pS24
      4.4.4 Synthesis of [^{18}F]-4-fluoroacetophenone with $Cu(OTf)_2$ elution pS24

5. Spectroscopic Data and Accompanying Data Tables pS26
   5.1 Synthesis of [^{18}F]-4-fluoroacetophenone with DMAP elution pS28
   5.2 Synthesis of [^{18}F]-4-fluorophenacylbromide with DMAP elution pS44
   5.3 Synthesis of [^{18}F]-4-fluoroacetophenone with $Cu(OTf)_2$ elution pS48

6. Substrate Scope Spectroscopic Data pS52
   6.1 4-[^{18}F]Fluoroacetophenone (**2**) pS52
   6.2 2-[^{18}F]Fluoromethylbenzoate **(4)** pS54
   6.3 4-[^{18}F]Fluorobenzonitrile **(5)** pS56
   6.4 4-[^{18}F]Fluoronitrobenzene **(6)** pS58
   6.5 4-[^{18}F]-2,4-Difluoroaniline **(7)** pS60
   6.6: 5-[^{18}F]Fluoroindole **(8)** pS62

7. Regression Analysis pS64

1. Materials and Methods

Reagents and solvents were purchased and used without further purification unless otherwise noted. HPLC grade acetonitrile, HPLC grade methanol, anhydrous sodium sulfate, potassium chloride, potassium bromide, potassium iodide, potassium dihydrogen phosphate, potassium hydrogen phosphate, potassium acetate, potassium hydroxide, sodium bicarbonate, and potassium carbonate were purchased from Fisher Scientific. Anhydrous *N*,*N*-dimethylformamide (DMF) and 4-acetylphenylboronic acid, pinacol ester were purchased from Acros Organics. Potassium trifluoromethanesulfonate was purchased from Oakwood Scientific. Copper(II) trifluoromethanesulfonate ($Cu(OTf)_2$) and 4-acetylphenylboronic acid, along with all other nitrogenous weak bases and preconditioning salts were purchased from Sigma-Aldrich. Sterile product vials were purchased from Hollister-Stier. QMA-light Sep-Paks were purchased from Waters Corporation. QMA-light Sep-Paks were conditioned with sequential flushes with 10 mL of ethanol, followed by 10 mL of 0.5 M preconditioning solution (*vide infra*), and 10 mL of sterile water prior to use.

2. Evaluation of Eluents and Preconditioning Reagents

2.1 Preparation of aqueous [^{18}F]Fluoride. All loading operations were conducted under an ambient atmosphere. Argon was used as a pressurizing gas during automated sample transfers. Fluorine-18 was produced via the $^{18}O(p,n)^{18}F$ nuclear reaction using a GE PETTrace cyclotron (40 µA beam for 2 min generated ca. 150 mCi of fluorine-18). Fluorine-18 was delivered to a TRACERLab FX_{FN} automated radiochemistry synthesis module in a 1.5 mL bolus of [^{18}O]water and collected in a sterile vial. Aqueous fluorine-18 produced in this way was further diluted with Milli-Q water to c.a. 1-3 mCi/mL prior to use in fluorine-18 elution studies.

2.2 [^{18}F]Fluoride elution studies method. Waters QMA-light Sep-Paks were washed sequentially with 10 mL ethanol, 10 mL 0.5 M preconditioning agent in water, and 10 mL Milli-Q water. 0.5 mL of aqueous [^{18}F]fluoride was passed through a QMA cartridge followed by 2 mL air, and the activity of the QMA cartridge was determined with a Capintec® dose calibrator. [^{18}F]fluoride was then eluted from the QMA cartridge into a 4mL vial with 0.5 mL eluent solution, followed by 2 mL of air. Activity of the 4 mL vial (eluate) and QMA cartridge (residual

[18F]fluoride) were determined with a Capintec dose calibrator. Activity data was used to calculate % fluoride recovery.

%[18F]Fluoride Recovery = Eluate Activity ÷ (Eluate Activity + Final QMA Activity)

2.3 Evaluation of Eluents and Preconditioning Reagents: Raw Data

2.3.1 [18F]Fluoride elution studies data: Copper Triflate as eluent

	Activity in microcuries (µCi)			
	Preconditioning Agent: 0.5M $NaHCO_3$			
$CuOTf_2$ concentration (M)	QMA initial	QMA final	eluate	^{18}F recovery (%)
0.01	409	311	90	22%
0.025	460	66	373	85%
0.05	447	20	415	95%
0.075	432	20	388	95%
0.1	334	10	317	97%
0.1 (MeCN)	430	317	107	25%
0.1 (MeOH)	421	220	203	48%

Table 2.3.1A: Elution of [18F]Fluoride with $Cu(OTf)_2$ after $NaHCO_3$ Preconditioning

	Activity in microcuries (µCi)			
	Preconditioning Agent: 0.5M KOTf			
$CuOTf_2$ concentration (M)	QMA initial	QMA final	eluate	^{18}F recovery (%)
0.01	416	188	217	54%
0.025	459	40	406	91%
0.05	441	20	407	95%
0.075	420	30	397	93%
0.1	326	40	284	88%
0.1 (MeCN)	408	286	114	29%
0.1 (MeOH)	446	160	272	63%

Table 2.3.1B: Elution of [18F]Fluoride with $Cu(OTf)_2$ after KOTf Preconditioning

	Activity in microcuries (µCi)			
	Preconditioning Agent: 0.5M Na_2SO_4			
$CuOTf_2$ concentration (M)	QMA initial	QMA final	eluate	^{18}F recovery (%)
0.01	447	99	330	77%
0.025	481	40	416	91%
0.05	436	30	396	93%
0.075	469	40	404	91%
0.1	353	20	315	94%
0.1 (MeCN)	419	311	110	26%
0.1 (MeOH)	440	209	229	52%

Table 2.3.1C: Elution of [^{18}F]Fluoride with $Cu(OTf)_2$ after Na_2SO_4 Preconditioning

2.3.2 [^{18}F]Fluoride elution studies data: KOH as eluent

	Activity in microcuries (µCi)			
	Preconditioning Agent= 0.5M $NaHCO_3$			
KOH concentration (M)	QMA initial	QMA final	eluate	^{18}F recovery (%)
0.02	534	75	451	84%
0.01	579	123	433	75%
0.0075	556	349	190	34%
0.005	547	423	116	21%
	Activity in microcuries (µCi)			
	Preconditioning Agent= 0.5M KOTf			
KOH concentration (M)	QMA initial	QMA final	eluate	^{18}F recovery (%)
0.02	546	375	169	31%
0.01	600	418	171	29%
0.0075	625	605	22	4%
0.005	591	592	0	0%
	Activity in microcuries (µCi)			
	Preconditioning Agent= 0.5M Na_2SO_4			
KOH concentration (M)	QMA initial	QMA final	eluate	^{18}F recovery (%)
0.02	830	67	739	89%
0.01	824	163	640	78%
0.0075	780	162	612	78%
0.005	780	300	484	62%

Table 2.3.2: Elution of [^{18}F]Fluoride with KOH after $NaHCO_3$, KOTf or Na_2SO_4 Preconditioning

2.3.3 [^{18}F]Fluoride elution studies data: Preconditioning studies with KOH as Eluent

Preconditioning Agent (as 0.5M aqueous solution)	Activity in microcuries (µCi) Eluent = 0.02M KOH			
	QMA initial	QMA final	eluate	^{18}F recovery (%)
NH_4HCO_3	1089	250	820	77%
NEt_4HCO_3	1160	282	830	75%
Na_2CO_3	1120	85	1035	92%
K_2CO_3	1138	70	1030	94%
KOH	1290	343	900	72%
KCl	1185	522	629	55%
KBr	1260	395	821	68%
KI	1160	249	895	78%
KH_2PO_4	2340	730	1498	67%
K_2HPO_4	1170	70	1085	94%
$NaHCO_2$	1175	822	340	29%
KOAc	1086	900	170	16%
PyridiniumOTs	1347	448	925	67%

Preconditioning Agent (as 0.5M aqueous solution)	Activity in microcuries (µCi) Eluent = 0.0075M KOH			
	QMA initial	QMA final	eluate	^{18}F recovery (%)
NH_4HCO_3	1040	885	160	15%
NEt_4HCO_3	1200	1100	85	7%
Na_2CO_3	1070	190	875	82%
K_2CO_3	1135	235	840	78%
KOH	960	298	635	68%
KCl	1110	1000	70	7%
KBr	1020	950	55	5%
KI	1100	890	209	19%
KH_2PO_4	1100	1030	69	6%
K_2HPO_4	1142	365	760	68%
$NaHCO_2$	1157	1115	17	2%
KOAc	1096	1076	30	3%
PyridiniumOTs	1090	766	300	28%

Table 2.3.3: Elution of [^{18}F]Fluoride with KOH after Different QMA Preconditioning Agents

2.3.4 [^{18}F]Fluoride elution studies data: Preconditioning studies with copper elution

We tested the impact of preconditioning agent on [^{18}F]fluoride recovery with aqueous $Cu(OTf)_2$, to see if this effect was unique for basic eluents. The results were less conclusive, as $Cu(OTf)_2$ led to very high % [^{18}F]fluoride recovery at most concentrations tested (within 0.025-0.1 M), but it was again observed that Na_2SO_4 led to greater % [^{18}F]fluoride recovery at the lowest concentration in water (0.01 M). Interestingly, a different preconditioning trend was observed with the poorer-performing methanolic and acetonitrile elution, where KOTf preconditioning promoted greater % [^{18}F]fluoride recovery than either $NaHCO_3$ or Na_2SO_4. This may be due to its *less* ionic character in an organic medium and/or lower solubility of these salts in organic solvents.

				^{18}F recovery (%) cartridge precond. agent		
**Entry**	**salt**	**eluent solvent**	**concentration (M)**	**KOTf**	**$NaHCO_3$**	**Na_2SO_4**
1	$Cu(OTf)_2$	water	0.01	54	22	77
2	$Cu(OTf)_2$	water	0.025	91	85	91
3	$Cu(OTf)_2$	water	0.05	95	95	93
4	$Cu(OTf)_2$	water	0.075	93	95	91
5	$Cu(OTf)_2$	water	0.1	88	97	94
6	$Cu(OTf)_2$	methanol	0.1	63	48	52
7	$Cu(OTf)_2$	acetonitrile	0.1	29	25	26

Table 2.3.4: Elution of [^{18}F]fluoride with $Cu(OTf)_2$ after Different QMA Preconditioning Agents

2.3.5 [^{18}F]Fluoride elution studies data: Weak bases as eluents

	Activity in microcuries (µCi)			
Eluent	Preconditioning Agent= 0.5M $NaHCO_3$			
(as 0.1M aqueous solution)	QMA initial	QMA final	eluate	^{18}F recovery (%)
pyridine	519	526	0	0%
aniline	870	890	0	0%
imidazole	820	810	0	0%
morpholine	830	493	291	35%
DABCO	820	366	430	52%
kryptofix	748	108	630	84%
ammonia	719	210	493	69%
ethanolamine	749	197	521	70%
methylamine	720	89	610	85%
diethylamine	740	65	654	88%
triethylamine	660	80	563	85%
DIPEA	663	71	584	88%
DBU	634	15	592	93%
4OMe pyridine	615	595	12	2%
lutidine	584	580	7	1%
collidine	577	534	28	5%
DMAP	621	149	453	73%

Table 2.3.5A: Elution of [^{18}F]Fluoride with Weak Bases after $NaHCO_3$ Preconditioning

	Activity in microcuries (µCi)			
Eluent	Preconditioning Agent= 0.5M KOTf			
(as 0.1M aqueous solution)	QMA initial	QMA final	eluate	^{18}F recovery (%)
pyridine	518	516	0	0%
aniline	830	810	0	0%
imidazole	820	840	0	0%
morpholine	798	738	50	6%
DABCO	810	725	70	9%
kryptofix	752	461	290	39%
ammonia	690	475	203	29%
ethanolamine	690	490	205	30%
methylamine	735	254	440	60%
diethylamine	691	123	523	76%
triethylamine	663	208	458	69%
DIPEA	654	211	436	67%
DBU	662	27	622	94%
4OMe pyridine	582	579	2	0%
lutidine	670	684	0	0%
collidine	601	593	8	1%
DMAP	592	390	205	35%

Table 2.3.5B: Elution of [^{18}F]Fluoride with Weak Bases after KOTf Preconditioning

	Activity in microcuries (μCi)			
Eluent	Preconditioning Agent= 0.5M Na_2SO_4			
(as 0.1M aqueous solution)	QMA initial	QMA final	eluate	^{18}F recovery (%)
pyridine	868	820	30	3%
aniline	960	940	20	2%
imidazole	970	497	438	45%
morpholine	910	280	599	66%
DABCO	900	265	633	70%
kryptofix	970	132	810	84%
ammonia	880	168	678	77%
ethanolamine	890	104	768	86%
methylamine	850	98	730	86%
diethylamine	820	73	741	90%
triethylamine	880	87	792	90%
DIPEA	842	69	758	90%
DBU	840	30	803	96%
4OMe pyridine	960	713	210	22%
lutidine	900	719	182	20%
collidine	940	245	687	73%
DMAP	791	134	637	81%

Table 2.3.5C: Elution of [^{18}F]Fluoride with Weak Bases after Na_2SO_4 Preconditioning

2.3.6 [^{18}F]fluoride elution studies data: 4-OMe Pyridine and Pyridine elution optimization studies

	Activity in microcuries (µCi)			
Preconditioning Agent	Eluent = 0.1M 4-OMe Pyridine			
(as 0.5M aqueous solution)	QMA initial	QMA final	eluate	^{18}F recovery (%)
$NaHCO_3$	534	521	0	0%
KOTf	518	517	0	0%
Na_2CO_3	495	487	0	0%
K_2CO_3	491	498	0	0%
K_2HPO_4	465	351	108	24%
Na_2SO_4	493	253	238	48%

	Activity in microcuries (µCi)			
Preconditioning Agent	Eluent = 0.1M Pyridine			
(as 0.5M aqueous solution)	QMA initial	QMA final	eluate	^{18}F recovery (%)
$NaHCO_3$	519	526	0	0%
KOTf	518	516	0	0%
Na_2CO_3	504	500	0	0%
K_2CO_3	514	495	0	0%
K_2HPO_4	502	480	20	4%
Na_2SO_4	498	407	70	15%

	Activity in microcuries (µCi)			
	Preconditioning Agent: 0.5M Na_2SO_4			
4-OMe pyridine conc. (M)	QMA initial	QMA final	eluate	^{18}F recovery (%)
0.1	493	253	238	48%
0.25	466	204	245	55%
0.5	445	205	232	53%
0.75	425	172	245	59%
1	429	139	270	66%

	Activity in microcuries (µCi)			
	Preconditioning Agent: 0.5M Na_2SO_4			
pyridine conc. (M)	QMA initial	QMA final	eluate	^{18}F recovery (%)
0.1	498	407	70	15%
0.25	417	323	99	23%
0.5	445	355	88	20%
0.75	449	343	91	21%
1	434	228	195	46%

Table 2.3.6: Elution of [^{18}F]Fluoride with Pyridine or 4-OMe-Pyridine

3. Synthesis of [^{18}F]-4-fluoroacetophenone *via* manual reactions

3.1 Preparation of [^{18}F]fluoride solution for manual syntheses. All loading operations were conducted under an ambient atmosphere. Argon was used as a pressurizing gas during automated sample transfers. Potassium [^{18}F]fluoride ([^{18}F]KF), was prepared using a TRACERLab FX_{FN} automated radiochemistry synthesis module (General Electric, GE). Fluorine-18 was produced via the $^{18}O(p,n)^{18}F$ nuclear reaction using a GE PETTrace cyclotron (40 μA beam for 2 min generated ca. 150 mCi of fluorine-18). Fluorine-18 was delivered to the synthesis module in a 1.5 mL bolus of [^{18}O]water and trapped on a QMA-light Sep-Pak (as [^{18}F]fluoride) to remove [^{18}O]water and other impurities. This was followed by elution of [^{18}F]fluoride into the reactor, followed by the addition of acetonitrile with/without phase transfer reagents (see below). Azeotropic drying was achieved by heating the reaction vessel to 100 °C and drawing vacuum for 6 min. The reaction vessel was then subjected to a stream of Ar and simultaneous vacuum draw for an additional 6 min at 100 °C. Following azeotropic drying, 6 mL of DMF was added to the dried reagent, and heated at 100 °C with stirring for 5 min. The resulting stock solution was cooled to 50 °C and transferred to a sterile vial for subsequent use in reactions. A 2 min bombardment typically led to a final stock activity of ~36-72 (6-12 mCi/mL) for the preparations below corresponding to 24-48% transfer efficiency based on starting activity (~150 mCi), with losses likely related to the efficiency of dissolving all of the [^{18}F]fluoride and transferring it out of the synthesis module. The amounts of [^{18}F]fluoride obtained were adequate for conducting the reactions described herein, although there is still scope for further optimization.

[^{18}F]KF/K_2CO_3/$K_{2.2.2}$: [^{18}F]Fluoride was eluted into the reaction vessel using 500 μL of 7mg/mL aqueous K_2CO_3. $K_{2.2.2}$ (15 mg) in MeCN (1 mL) was added to the reaction vessel, and the resulting solution was dried by azeotropic distillation to provide dry [^{18}F]KF/K_2CO_3/ $K_{2.2.2.}$

[^{18}F]KF/KOTf: [^{18}F]Fluoride was eluted into the reaction vessel using 500 μL of 10mg/mL aqueous KOTf. MeCN (1 mL) was added to the reaction vessel, and the resulting solution was dried by azeotropic distillation to provide dry [^{18}F]KF/KOTf.

[^{18}F]DMAPH$^+$F$^-$: [^{18}F]Fluoride was eluted into the reaction vessel using 500 μL of 0.1 M aqueous dimethylaminopyridine (DMAP). MeCN (1 mL) was added to the reaction vessel, and the resulting solution was dried by azeotropic distillation to provide dry [^{18}F]DMAPH$^+$F$^-$.

3.2 Manual Synthesis Method

Stock solutions of acetylphenylboronic acid, pinacol ester (APBpin) or other Bpin-appended precursor, copper (II) trifluoromethanesulfonate ($Cu(OTf)_2$), dimethylaminopyridine (DMAP), and/or other additive(s) in DMF were prepared immediately prior to the start of the reaction. Aliquots of these solutions were used to carry out subsequent [^{18}F]fluorination reactions. In a typical reaction, 660 µL anhydrous DMF was mixed with 40 µL $Cu(OTf)_2$ solution (8 µmol, 0.16 equiv, 200 mM), 100 µL Bpin solution (50 µmol, 1 equiv, 500mM) and 100 µL DMAP solution (50 µmol, 1 equiv, 500 mM) in a colorless borosilicate 4 mL scintillation vial. The reaction vial was sealed under ambient atmosphere with a PTFE/Silicone septum cap, and 100 µL of [^{18}F]fluoride stock (150-500 µCi) was added to the reaction vial through the septum *via* syringe. Additional anhydrous DMF was added (as required) to bring the total solution volume to 1000 µL. The vial was then heated in an aluminum block (Chemglass Part# CG-1991-04) without stirring at 110 ºC for 20 min. After 20 min, the reaction was allowed to cool to room temperature.

3.3 Radiolabeling Optimization Screens

4-acetylphenylboronic acid, pinacol ester (APBpin) was used as the [^{18}F]-fluorination substrate for all chemistry optimization screens. The reaction scheme, as well as accompanying tables in each subsection describes the reaction conditions employed; with **bold** typeface in the reaction scheme denoting the variable tested in each case. All reactant values are expressed in µmol quantities for brevity and simplicity. Red typeface denotes the [^{18}F]fluoride source used.

S3.3.1 Reagent Loading Screen

Stock solutions of 4-acetylphenylboronic acid, pinacol ester (APBpin, 100 µmol/mL), $Cu(OTf)_2$ (200 µmol/mL) and DMAP (500 µmol/mL) in anhydrous DMF were prepared 15-30 min prior to the start of the reaction(s). Appropriate volumes of the reagent solutions were added to colorless borosilicate 4 mL scintillation vials *via* disposable pipette to obtain the desired reagent quantity/ratios (see Table 3.3.1 below for exact reagent quantities), and additional anhydrous DMF was added to bring the total volume in the vials to 900 µL. The reaction vials were sealed under ambient atmosphere with a PTFE/Silicone septum cap, and 100 µL of [^{18}F]KF/KOTf DMF stock (approx. 500 µCi) was added to the reaction vial through the septum *via* syringe. The vial was then heated in an aluminum block (Chemglass Part# CG-1991-04) without stirring at 110 ºC for 20 min. The reaction was then allowed to cool, and once the reaction mixture was sufficiently cool to handle, Radio-TLC analysis was conducted to determine radiochemical conversion (RCC in %). Crude reaction mixture was spotted onto standard silica coated glass plates and developed with 1:1 hexane/ethyl acetate in a glass TLC chamber. The RCC was determined by dividing the integrated area under the fluorinated product spot by the total integrated area of all peaks on the TLC plate.

Reagent loading screen

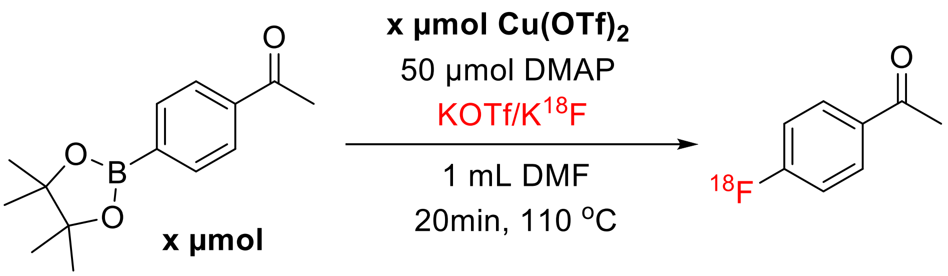

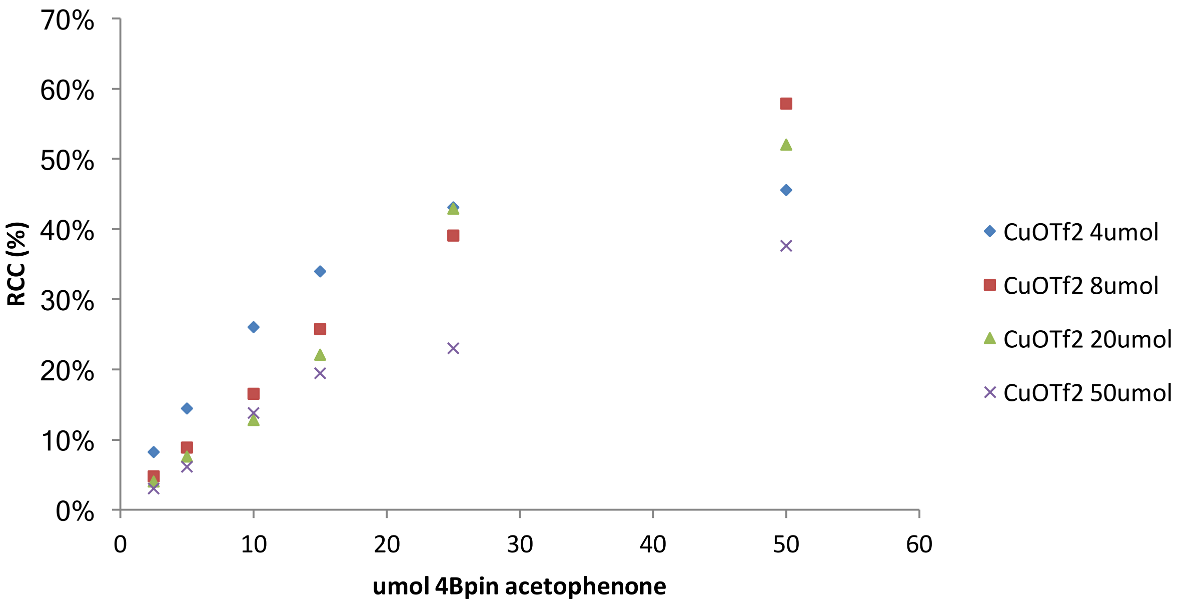

Chart S3.3.1: Reagent loading screen

	RCC (%)					
**Sample #**	**1**	**2**	**3**	**4**	**5**	**6**
DMAP μmol	50	50	50	50	50	50
APBpin μmol	2.5	5	10	15	25	50
**$Cu(OTf)_2$ 4 μmol**	**8.2%**	**14.4%**	**26.0%**	**34.0%**	**43.1%**	**45.6%**
**$Cu(OTf)_2$ 8 μmol**	**4.8%**	**8.9%**	**16.5%**	**25.8%**	**39.1%**	**57.9%**
**$Cu(OTf)_2$ 20 μmol**	**4.0%**	**7.6%**	**12.8%**	**22.1%**	**42.9%**	**52.0%**
**$Cu(OTf)_2$ 50 μmol**	**3.0%**	**6.1%**	**13.8%**	**19.5%**	**23.0%**	**37.6%**

Table 3.3.1: Reagent loading screen

S3.3.2 Order of Addition Screen (Manual)

Stock solutions of 4-acetylphenylboronic acid, pinacol ester (APBpin, 100 µmol/mL), $Cu(OTf)_2$ (200 µmol/mL) and DMAP (500 µmol/mL) in anhydrous DMF were prepared 60 min prior to the start of the reaction(s). Sixteen colorless borosilicate 4 mL scintillation vials were charged with 260 µL anhydrous DMF *via* pipette. Aliquots of APBpin (50 µmol, 500 µL), $Cu(OTf)_2$ (8 µmol, 40 µL), DMAP (50 µmol, 100 µL), and/or [^{18}F]KF/KOTf DMF stock (approx. 500 µCi, 100 µL) were then added *via* pipette, with each vial containing a different mixture of reagents (i.e, specific reagents were added to some vials, but not others; there are coincidentally sixteen different reagent mixture variations (2^4) that can be made from four different reagents). Table 3.3.2 shows which reagents were added at this stage of the reaction and which were not (**X** = added; **O** = not added). Following reagent addition, the reaction vials were sealed under ambient atmosphere with a PTFE/Silicone septum cap and heated in an aluminum block (Chemglass Part# CG-1991-04) without stirring at 110 ºC for 20 min. The reaction was allowed to cool to room temperature and the reagents not added previously were added to the cooled reaction solution (i.e, all vials now contained equivalent aliquots of all four reagents). The vials were re-sealed under ambient atmosphere with a PTFE/Silicone septum cap and heated again in an aluminum block (Chemglass Part# CG-1991-04) without stirring at 110 ºC for 20 min. After this second round of heating, the reaction was allowed to cool, and once the reaction mixture was sufficiently cool to handle, Radio-TLC analysis was conducted to determine radiochemical conversion (RCC in %). Crude reaction mixture was spotted onto standard silica coated glass plates and developed with 1:1 hexane/ethyl acetate in a glass TLC chamber. The RCC was determined by dividing the integrated area under the fluorinated product spot by the total integrated area of all peaks on the TLC plate.

Order of Addition screen (Manual)

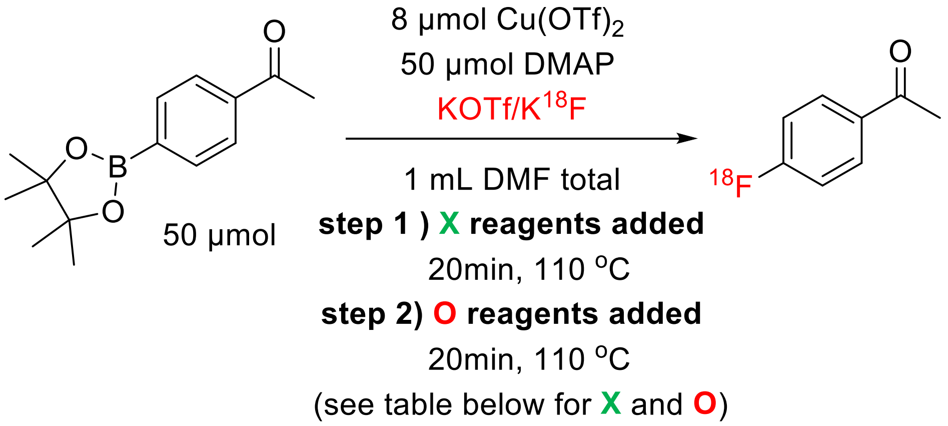

Procedure:
step 1) Added X; stirred @ 110C for 20 min; cooled;

step 2) added O; stirred @ 110C for 20 min; cooled; radio TLC

DMAP	X	O	X	X	O	O	X	O
$Cu(OTf)_2$	X	X	O	X	X	O	O	O
APBpin	X	X	X	O	O	X	O	O
[^{18}F]KF/KOTf	X	X	X	X	X	X	X	X
**RCC (%)**	**59.0%**	**4.2%**	**65.5%**	**40.4%**	**11.9%**	**63.1%**	**54.3%**	**63.7%**
DMAP	X	O	X	X	O	O	X	O
$Cu(OTf)_2$	X	X	O	X	X	O	O	O
APBpin	X	X	X	O	O	X	O	O
[^{18}F]KF/KOTf	O	O	O	O	O	O	O	O
**RCC (%)**	**4.2%**	**4.0%**	**67.2%**	**45.2%**	**23.8%**	**62.6%**	**60.4%**	**57.8%**

Table 3.3.2: Order of Addition screen (Manual)

S3.3.3 Order of Addition Screen (Automated)

We conducted automated optimization and order of addition studies using a TRACERLab FX_{FN} synthesis module. As the radiochemical yield of a radiolabeling reaction is dependent on both recovery during [^{18}F]fluoride elution and the reaction RCC, three preconditioning strategies were tested separately due to their different [^{18}F]fluoride recovery yields, but unknown effects on RCC (Table 3.3.3, entries 1-3). The highest RCC (5%) was observed with $NaHCO_3$ preconditioning (entry 1). In addition to low RCC, KOTf preconditioning (entry 2) also led to unacceptably low [^{18}F]fluoride recovery and hence was not explored further. Given the potential improvements offered by QMA preconditioning with Na_2SO_4, we also tested this possibility (entry 3). Unfortunately, in this case, there was no product formation, likely due to deactivation of the catalyst via the coordination of SO_4^{2-} to Cu^{2+}. This result was not entirely unexpected, as it is in line with our previous findings that $CuSO_4$ is an inadequate catalyst for this chemistry (see main manuscript). This finding demonstrates the need to carefully consider fluoride processing when designing and/or optimizing late-stage fluorination approaches.

The order of reagent addition was then re-evaluated to examine if it impacts the reactivity. Order of addition was examined for both the manual reactions (see Section 3.3.2 above) and automated reactions performed in the TRACERLab FX_{FN} synthesis module, using $NaHCO_3$ as the preconditioning agent. Anhydrous [^{18}F]fluoride was pre-dissolved in either $Cu(OTf)_2$ or arylBpin DMF solutions (110 °C, 5 min) prior to addition of the other reagents and radiolabeling (140 °C, 20 min). Pre-dissolving [^{18}F]fluoride with the $Cu(OTf)_2$ solution again led to low RCCs of 2-4% (Table 3.3.3, entries 4 and 5), but pre-dissolving [^{18}F]fluoride with arylBpin 1 led to an RCC of 19% (Table 3.3.3, entry 6). Conducting this reaction using the optimized reagent ratio (see Figure 2b of the main manuscript) further increased the RCC to 38% and 45%, when the reaction was carried out at 140 °C and 110 °C, respectively (Table 3.3.3, entries 7 and 8). Interestingly when conducting manual reactions, pre-mixing and heating $Cu(OTf)_2$, substrate, and DMAP prior to the addition of [^{18}F]fluoride also led to significant yield suppression, while simultaneous mixing of all the reactants with [18F]fluoride stock solution did not (see Section 3.3.2 above), suggesting that dissolution of [^{18}F]fluoride is a critical step in this synthesis, and must occur prior to the addition of [^{18}F]fluoride or substrate to $Cu(OTf)_2$. Mechanistic studies into these effects are currently underway.

Entry	Reaction conditions	Precond. agent	Reaction temp (°C)	Target Water Activity (mCi)	Final Dose Activity (mCi)	RCC (%)
	**Unoptimized conditions**[(a)]					
1	^{18}F mixed with all reactants	$NaHCO_3$	110	120	12	**5**
2	^{18}F mixed with all reactants	KOTf	140	150	12	**2**
3	^{18}F mixed with all reactants	Na_2SO_4	140	150	37	**0**
4	**1:** ^{18}F mixed with $CuOTf_2$<br>**2:** Precursor, DMAP added	$NaHCO_3$	**1:** 110<br>**2:** 140	160	41	**2**
5	**1:** ^{18}F mixed with $CuOTf_2$<br>**2:** Precursor added	$NaHCO_3$	**1:** 110<br>**2:** 140	150	38	**4**
6	**1:** ^{18}F mixed with Precursor<br>**2:** $CuOTf_2$, DMAP added	$NaHCO_3$	**1:** 110<br>**2:** 140	150	39	**19**
	**Optimized conditions**[(b)]					
7	**1:** ^{18}F mixed with Precursor<br>**2:** $CuOTf_2$, DMAP added	$NaHCO_3$	**1:** 110<br>**2:** 140	150	42	**38**
8	**1:** ^{18}F mixed with Precursor<br>**2:** $CuOTf_2$, DMAP added	$NaHCO_3$	**1:** 110<br>**2:** 110	150	47	**45**

Table 3.3.3: Automated synthesis results using aqueous dimethylaminopyridine as the eluent and order of addition as a variable. (a) **Unoptimized conditions:** 4-dimethylaminopyridine (Eluent + 50 μmol, 2.5 equiv, 100 mM) copper (II) trifluoromethanesulfonate (20 μmol, 2 equiv, 20 mM), and 4-acetylphenylboronic acid, pinacol ester (40 μmol, 2 equiv, 40 mM) in a total volume of 1 mL anhydrous dimethylformamide; (b) **Optimized conditions:** 4-dimethylaminopyridine (Eluent + 50 μmol, 1 equiv, 100 mM), copper (II) trifluoromethanesulfonate (8 μmol, 0.16 equiv, 16 mM), 4-acetylphenylboronic acid, pinacol ester (50 μmol, 1 equiv, 100 mM) in a total volume of 1 mL anhydrous dimethylformamide. (Step **1** = 5 min; Step **2** = 20 min).

3.4 Substrate Scope

Radiofluorination of several Bpin substrates was conducted as described in Section 3.2, however overall reagent loading was halved (see figure below). RCC was determined using radioTLC (in 1:1 hexanes: ethyl acetate eluent) and identity was determined using HPLC (see Section 6 for raw data).

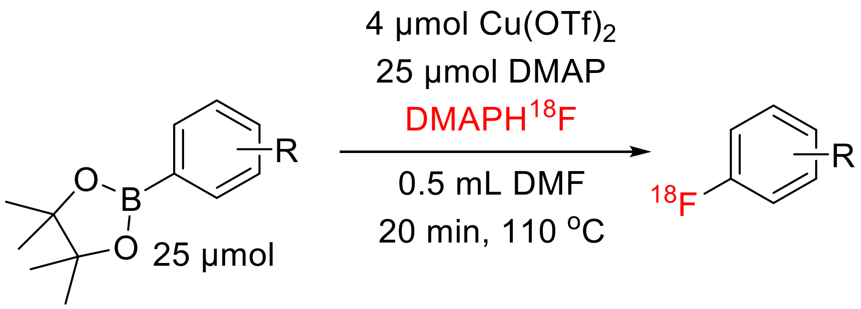

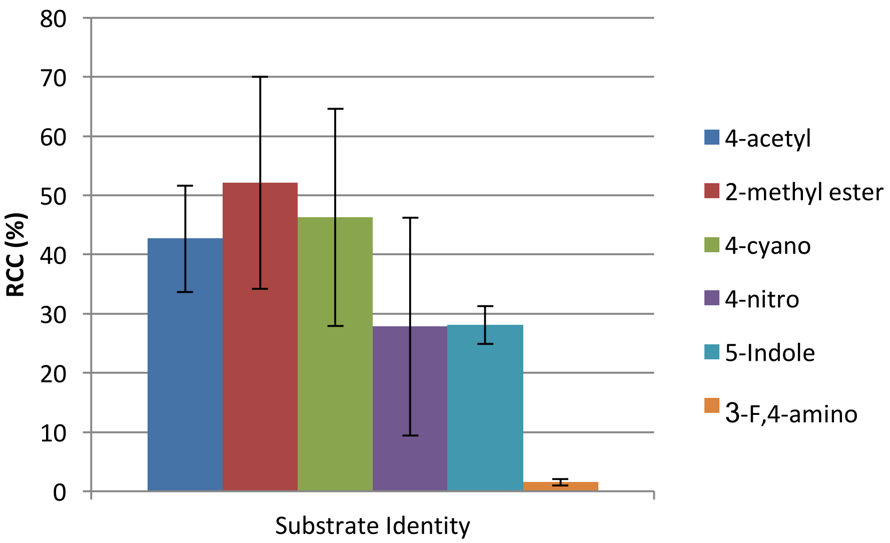

Chart S3.4: Substrate Screen

	RCC%					
Substrate (R =)	4-acetyl (**2**)	2-methyl ester (**4**)	4-cyano (**5**)	4-nitro (**6**)	3-F,4-amino (**7**)	5-Indole (**8**)
	52.3	51.8	42.4	19.9	1.3	31.0
	41.1	70.1	66.2	48.9	2.1	28.5
	34.6	34.3	30.2	14.7	1.2	24.7
Mean	43	52	46	28	1.5	28
SD	9	18	18	18	0.5	3

Table 3.4: Substrate Screen

4. Synthesis of [^{18}F]fluoroacetophenone in a TRACERlab FX_{FN} Synthesis Module.

4.1 General Considerations: Fluorine-18 was produced via the $^{18}O(p,n)^{18}F$ nuclear reaction using a GE PETTrace cyclotron (40 μA beam for 2 min generated ca. 150 mCi of fluorine-18 as measured by synthesis module detector). The [^{18}F]fluoride was delivered to a TRACERLab FX_{FN} automated radiochemistry synthesis module in a 1.5 mL bolus of [^{18}O]water and collected in a sterile vial. Fluorine-18 containing target water was then reprocessed and used for radiofluorination reactions according to methods described in sections 4.4-4.6. All synthesis module loading operations were conducted under an ambient atmosphere. Argon was used as a pressurizing gas during automated sample transfers. Total recovered activity at the end of synthesis was measured with a Capintec® dose calibrator.

4.2 Thin Layer Chromatography (TLC) Analysis: Radio-TLC analysis was conducted to determine % RCC using a Bioscan AR-2000 TLC scanner. Crude reaction mixture (undiluted) was spotted onto standard silica coated glass plates and developed with 1:1 hexane/ethyl acetate in a glass TLC chamber. The RCC was determined by dividing the integrated area under the fluorinated product spot by the total integrated area of the TLC plate.

4.3 HPLC analysis

Radio-HPLC analyses were conducted using a Shimadzu LC-2010A HT system equipped with a Bioscan B-FC-1000 radiation detector. To prepare samples for HPLC analysis, approx. 100 µL crude reaction mixture was added directly into HPLC vials without further dilution. To confirm identity, crude reaction mixture was spiked with 1 mg/mL 4-fluoroacetophenone in acetonitrile (typically 50 µL standard solution was added to 100 µL crude reaction mixture and briefly agitated). Eluent systems and columns used for HPLC analysis are described below.

HPLC Condition A.

Condition: 40 % MeCN in H_2O, 10mM NH_4OAc pH: 6.1

Flow Rate: 1 mL/min

Column: Phenomenex® Luna C-8 Column 150 x 4.6 mm. 3 µm.

HPLC Condition B.

Condition: 40 % MeCN in H_2O, 10mM NH_4OAc pH: 6.1

Flow Rate: 1 mL/min

Column: Waters® Spherisorb C-8 Column 150 x 4.6 mm. 5 µm.

HPLC Condition C.

Condition: 40 % MeCN in H_2O, 10mM NH_4OAc pH: 6.1

Flow Rate: 4 mL/min

Column: Phenomenex® Luna C-18 Column 250 x 10 mm. 5 µm.

HPLC Condition D.

Condition: 40 % MeCN in H_2O, 10mM NH_4OAc pH: 6.1

Flow Rate: 2 mL/min

Column: Waters® Spherisorb C-8 Column 150 x 4.6 mm. 5 µm.

HPLC Condition E.

Condition: 5-95 % MeCN gradient (time = 0-20 min), then held at 5% MeCN (time = 20-25 min). Water contained 0.1% trifluoroacetic acid, MeCN contained no additives.

Flow Rate: 2 mL/min,

Column: Waters® Spherisorb C-8 Column 150 x 4.6 mm. 5 µm.

4.4 General synthetic procedures in a TRACERLab FX_{FN} synthesis module

4.4.1 Synthesis of [^{18}F]-4-fluoroacetophenone ([^{18}F]FAP) with DMAP elution

Waters QMA-light cartridges was sequentially rinsed with 10 mL ethanol, 10 mL 0.5M preconditioning salt, and 10 mL water prior to use. Dry [^{18}F]DMAP●HF was produced by trapping ^{18}F from target water on the QMA cartridge followed by elution with 0.5 mL aqueous DMAP (50 µmol, 1 equiv, 100 mM) (vial 1) and azeotropic drying with 1 mL acetonitrile (vial 2) at 100 °C. The reactor housing the dry [^{18}F]fluoride was cooled to 50 °C with compressed air and the reactants (vial 3 and/or 4) in a total volume of 1 mL of anhydrous DMF were added to the reactor by applying Argon push gas through the valve containing the reagent solution. All open valves leading out of the reactor were then closed and the mixture was stirred at either 110 or 140 °C for 20 min. Under optimized conditions, the reactant ratios used were: $Cu(OTf)_2$ (8 µmol, 0.16 equiv), 4-acetylphenylboronic acid, pinacol ester (50 µmol, 1 equiv), DMAP (50 µmol, 1 equiv). In several instances, one or two of the reactants (vial 3) in 0.5 mL anhydrous DMF were added to the dry [^{18}F]fluoride first, and "pre-stirred" with the [^{18}F]fluoride for 5 min at 110 °C, cooled to 50 °C, and finally mixed with the remaining reactant(s) (vial 4) in 0.5 mL DMF. The (complete) reactant mixture was then stirred at either 110 or 140 °C for 20 min. The reactor was then cooled to 50 ºC and 5 mL of DMF was added to the reactor. The mixture was stirred ~2 min and was transferred to an 8 mL sterile product vial with Argon push gas. The product vial was transferred out of the synthesis module in a lead pig. Total activity of recovered material was measured using a Capintec dose calibrator. Radiochemical conversion (RCC) was determined with radio-TLC (Eluent = 1:1 hexanes: ethyl acetate). Identity of the product was confirmed with HPLC. The preconditioning salts and eluents used, as well as the order of addition and amounts of reactants, and radiochemical conversions are detailed in Table 5A. Activities at various timepoints during synthesis as well as radiochemical conversions are detailed in Table 5A. Spectroscopic data (radio-TLC and HPLC spectra) and HPLC conditions (column, flow rate, and eluent used) are located in section 5.1.1-5.1.10.

4.4.2 Synthesis of [^{18}F]-4-Fluorophenacyl Bromide ([^{18}F]FPB) with DMAP Elution

Waters QMA-light cartridge was sequentially rinsed with 10 mL ethanol, 10 mL 0.5M preconditioning salt, and 10 mL water prior to use. Dry [^{18}F]DMAP●HF was produced by trapping ^{18}F from target water on the QMA cartridge followed by elution with 0.5 mL aqueous DMAP (50 µmol, 1 equiv, 100 mM) (vial 1) and azeotropic drying with 1 mL acetonitrile (vial 2) at 100 °C. The reactor housing the dry [^{18}F]fluoride was cooled to 50 °C with compressed air, and then a solution (vial 3) containing 4-acetylphenylboronic acid, pinacol ester (50 µmol, 1 equiv) in 500 µL anhydrous DMF was added to the reactor using Ar push gas. The reactor was heated to 110 °C for 5 min and subsequently cooled to 50 °C with compressed air. A second solution (vial 4) containing $Cu(OTf)_2$ (8 µmol, 0.16 equiv) and DMAP (50 µmol, 1 equiv) in 500 µL anhydrous DMF was added to the reactor using Ar push gas, heated to 110 °C for 20 min and subsequently cooled to 50 °C with compressed air. Finally, a third solution (vial 5) containing methanesulfonic acid (1000 µmol, 20 equiv) and *N*-bromosuccinimide (150 µmol) in 1000 µL anhydrous acetonitrile was added to the reactor using Ar push gas, heated to 110 °C for 20 min and subsequently cooled to 50 °C with compressed air. 5 mL of DMF was added to the reactor, the mixture was stirred for ~2 min and was then transferred to an 8 mL sterile product vial with Argon push gas. Total activity of recovered material was measured using a Capintec dose calibrator. Radiochemical conversion (RCC) to [^{18}F]fluorinated organics was determined with radio-TLC (Eluent = 1:1 hexanes: ethyl acetate). The identity and ratio of organic [^{18}F]fluorinated products in the reaction mixture, which included [^{18}F]-4-fluoroacetophenone ([^{18}F]FAP), [^{18}F]-4-fluorophenacyl bromide ([^{18}F]FPB), and [^{18}F]-4-fluorophenacyl dibromide ([^{18}F]FPDB), was determined using HPLC. Activities at various timepoints during synthesis as well as radiochemical conversions are detailed in Table 5B. The relative percentages of [^{18}F]FAP, [^{18}F]FPB, and [^{18}F]FPDB are detailed in Table 5C. Spectroscopic data (radio-TLC and HPLC spectra) and HPLC conditions (column, flow rate, and eluent used) are located in section 5.2.1.

4.4.3 Synthesis of [^{18}F]-4-Fluorophenacyl Bromide ([^{18}F]FPB) with DMAP Elution: in-box purification and reformulation.

Waters QMA-light cartridge was sequentially rinsed with 10 mL ethanol, 10 mL 0.5M preconditioning salt, and 10 mL water prior to use. Dry [^{18}F]DMAP●HF was produced by trapping ^{18}F from target water on the QMA cartridge followed by elution with 0.5 mL aqueous DMAP (50 µmol, 1 equiv, 100 mM) (vial 1) and azeotropic drying with 1 mL acetonitrile (vial 2) at 100 °C. The reactor housing the dry [^{18}F]fluoride was cooled to 50 °C with compressed air, and then a solution (vial 3) containing 4-acetylphenylboronic acid, pinacol ester **BPin-1** (50 µmol, 1 equiv) in 500 µL anhydrous DMF was added to the reactor using Ar push gas. The reactor was heated to 110 °C for 5 min and subsequently cooled to 50 °C with compressed air. A second solution (vial 4) containing $Cu(OTf)_2$ (8 µmol, 0.16 equiv) and DMAP (50 µmol, 1 equiv) in 500 µL anhydrous DMF was added to the reactor using Ar push gas, heated to 110 °C for 20 min and subsequently cooled to 50 °C with compressed air. Finally, a third solution (vial 5) containing methanesulfonic acid (1000 µmol, 20 equiv) and *N*-bromosuccinimide (150 µmol, 3 equiv) in 1000 µL anhydrous acetonitrile was added to the reactor using Ar push gas, heated to 110 °C for 20 min and subsequently cooled to 50 °C with compressed air. A solution (vial 6) containing KOH (750 µmol, 15 equiv) in 2 mL of water was added to the reactor to neutralize excess acid, and the mixture was loaded onto a semi-preparative column (HPLC condition C). The [^{18}F]FPB product peak was collected (22.1-24.0 min) into a dilution flask containing 50 mL of water. The diluted product was then passed through a Waters C18 1cc vac cartridge, and the cartridge was rinsed with 10 mL of water (vial 9). Product trapped on the cartridge was eluted with 0.5 mL ethanol (vial 8), followed by 4.5 mL sterile saline buffer into a collection flask. The formulated product was then transferred to an 8 mL sterile product vial with Argon push gas. Total activity of recovered material was 13 mCi as measured using a Capintec dose calibrator. The identity (matched with co-injected FPB standard), specific activity (8,097 Ci/mmol) and radiochemical purity (99%) were determined using HPLC (condition D). HPLC data and HPLC conditions (column, flow rate, and eluent used) are located in section 5.2.2.

4.4.4 Synthesis of [^{18}F]-4-Fluoroacetophenone ([^{18}F]FAP) with $Cu(OTf)_2$ Elution

Waters QMA-light cartridge was sequentially rinsed with 10 mL ethanol, 10 mL 0.5M potassium trifluoromethanesulfonate, and 10 mL water. Dry [^{18}F]$Cu(OTf)_2$●F^- was produced by trapping ^{18}F from target water on the QMA cartridge followed by elution with 500 µL of aqueous

(5.3.1) or methanolic (5.3.2) $Cu(OTf)_2$ (50 µmol, 6.25 equiv, 100 mM, vial 1), and azeotropic drying with 1 mL acetonitrile (vial 2) at 100 °C. The reactor housing the dry [^{18}F]fluoride was cooled to 50 ^{0}C with compressed air and a solution containing 4-acetylphenylboronic acid (8 µmol, 1 equiv, 4 mM), and pyridine (1000 µmol, 125 equiv, 500 mM) in 2 mL anhydrous DMF (vial 3) was added to the reactor by applying Ar push gas through the vial containing the reagent solution. All open valves leading out of the reactor were then closed and the mixture was stirred for 20 min at 140 ºC. The mixture was cooled to 50 ºC with compressed air cooling and 5 mL of DMF was added to the reactor. The mixture was stirred for ~2 min and was transferred to an 8 mL sterile product vial with Ar push gas. The product vial was transferred out of the synthesis module in a lead pig. Total activity of recovered material was measured using a Capintec dose calibrator. Radiochemical conversion (RCC) was determined with radio-TLC (Eluent = 1:1 hexanes: ethyl acetate). Identity of the product was confirmed with HPLC. The activities at various timepoints during synthesis and radiochemical conversions are detailed in Table 5B. Spectroscopic data (radio-TLC and HPLC spectra) and HPLC conditions (column, flow rate, and eluent used) are located in section 5.3.1 and 5.3.2.

5. Spectroscopic data and accompanying data tables

Entry	QMA preparation Preconditioning Salt (0.5 M aq. soln)	μmol reagents used			Order of reagent addition		reaction temperature (°C)	RCC (%)
		CuOTf X	AP-Bpin O	DMAP[1] #	"pre-stirring" (5 min, 110 °C)	reaction (20 min, X °C)		
5.1.1	$NaHCO_3$	40	20	n/a	n/a	X, O	140	**5**
5.1.2	Na_2SO_4	40	20	n/a	n/a	X, O	140	**0**
5.1.3	KOTf	40	20	n/a	n/a	X, O	140	**2**
5.1.4	$NaHCO_3$	40	20	n/a	X	O	140	**2**
5.1.5	$NaHCO_3$	40	20	20	X	O, #	140	**4**
5.1.6	$NaHCO_3$	40	20	20	O	X, #	140	**19**
5.1.7	$NaHCO_3$	8	50	50	O	X, #	140	**38**
5.1.8	Na_2SO_4	8	50	50	O	X, #	140	**2**
5.1.9	$NaHCO_3$	8	50	50	O	X, #	110	**45**
5.1.10	Na_2SO_4	8	50	50	O	X, #	110	**0**

Table 5A: Reaction conditions used in syntheses 5.1.1 – 5.1.10

Entry	Target water[2] (2 min beam)	Activity at various stages of synthesis (mCi)			RCC (%)
		After elution[2]	After Azeotropic Drying[2]	Final activity in dose vial[3]	
5.1.1	120	50	25	12	5
5.1.2	150	140	90	37	0
5.1.3	150	45	30	12	2
5.1.4	160	140	90	41	2
5.1.5	150	120	100	38	4
5.1.6	150	90	50	39	19
5.1.7	150	80	65	42	38
5.1.8	165	145	80	34	2
5.1.9	150	100	70	47	45
5.1.10	160	155	110	42	0
5.2.1	150	90	70	30	26
5.3.1	150	140	65	19[4]	10
5.3.2	150	50	25	16	12

Table 5B: Activity (mCi) during various stages of synthesis

[1] This is DMAP that was added **in addition to** the DMAP present from the eluent in all syntheses 5.1. It should be noted, however, that an unknown fraction of the DMAP present from the eluent could be lost during $[^{18}F]$fluoride reprocessing and/or azeotropic evaporation (thus necessitating this "extra" DMAP).
[2] Readouts are from uncalibrated TRACERLab detectors and should be used as a rough guide only.
[3] Final activities are measured in a Capintec dose calibrator.
[4] In 5.3.1, a significant amount of product was lost on its way to the product vial due to a leaking line.

Entry	Ratio of [^{18}F]fluorinated organics (%)						RCC (to all organics) (%)
	[^{18}F]FAP		[^{18}F]FPB		[^{18}F]FPDB		
	Det. Resp.[5]	%	Det. Resp.	%	Det. Resp.	%	
5.2.1	11897	7	114432	69	40260	24	26

Table 5C: Ratio of [^{18}F]fluorinated organics (%) in synthesis 5.2.1

[5] Det. Resp. = HPLC RAD detector response (in mA). Ratio of [^{18}F]fluorinated products (expressed as %) was determined by comparing HPLC detector response values of FAP, FPB, and FPDB . Overall RCC (of all three products vs. "inorganic" fluoride) was determined using radio TLC and is shown in the rightmost column.

5.1 Synthesis of [^{18}F]-4-fluoroacetophenone with DMAP elution

5.1.1

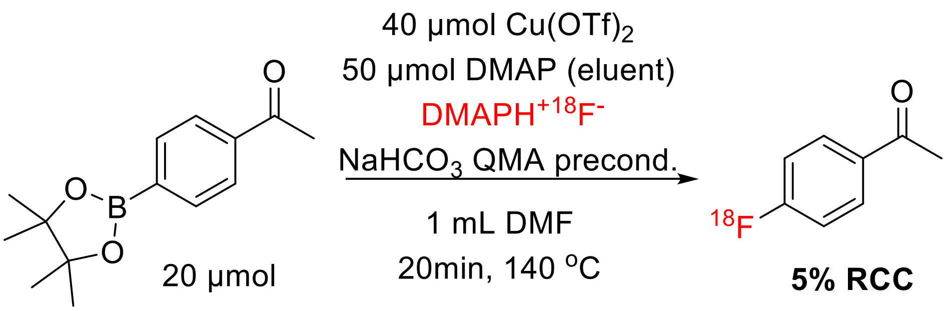

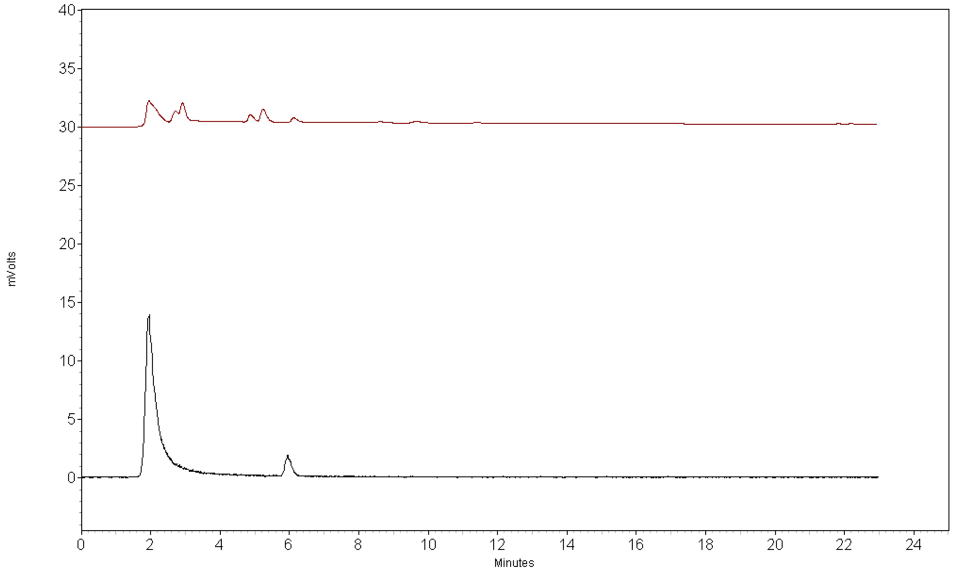

Figure 5.1.1A: HPLC spectra of crude reaction mixture from 5.1.1. RAD trace (black, bottom) and UV trace (256 nm, red, top). HPLC conditions A.

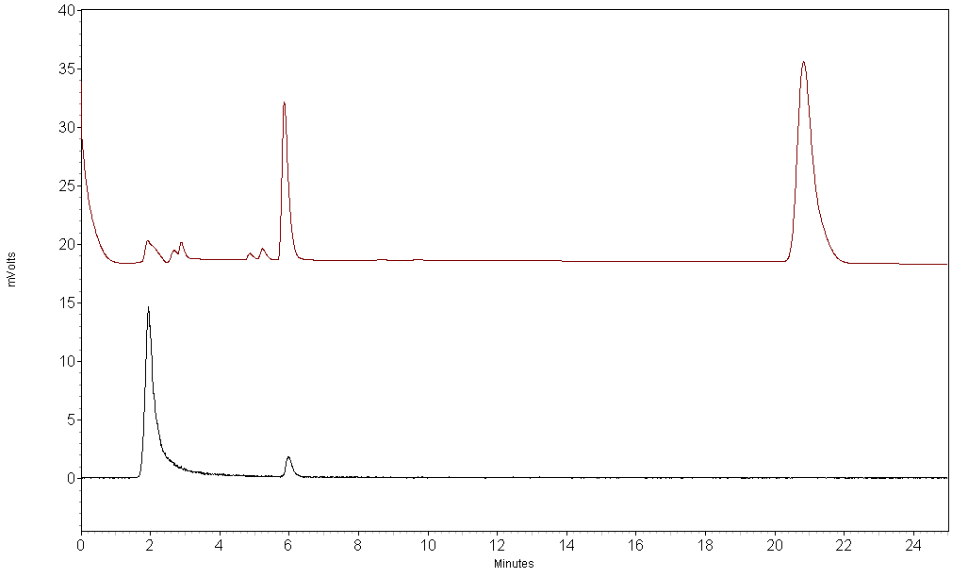

Figure 5.1.1B: HPLC spectra of crude reaction mixture from 5.1.1. spiked with 4-fluoroacetophenone reference standard. RAD trace (black, bottom) and UV trace (256 nm, red, top). HPLC conditions A.

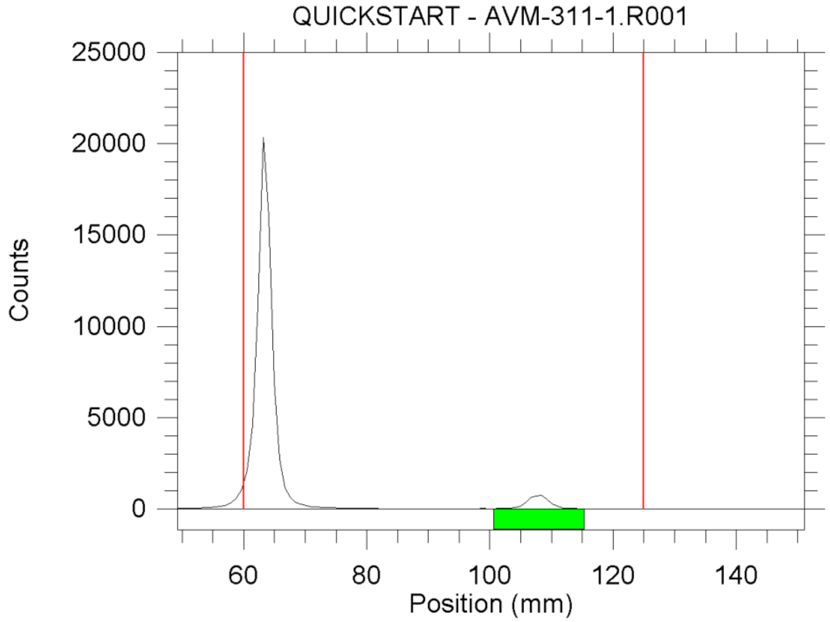

Figure 5.1.1C: Radio-TLC spectrum of the crude reaction mixture from 5.1.1

5.1.2

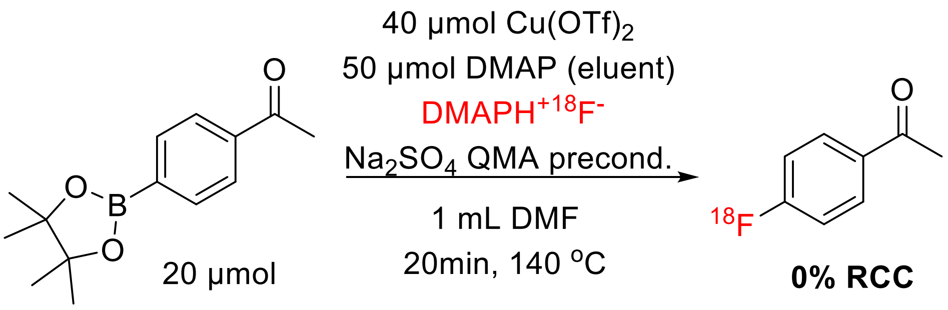

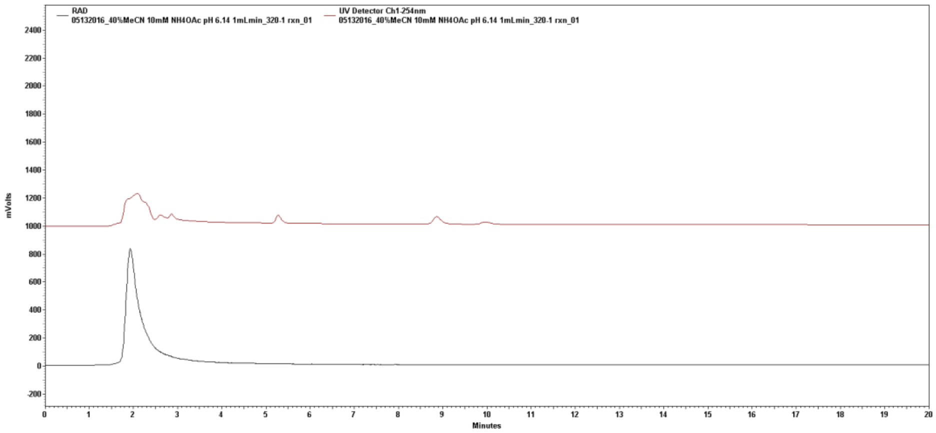

Figure 5.1.2A: HPLC spectra of crude reaction mixture from 5.1.2. RAD trace (black, bottom) and UV trace (256 nm, red, top). HPLC conditions A.

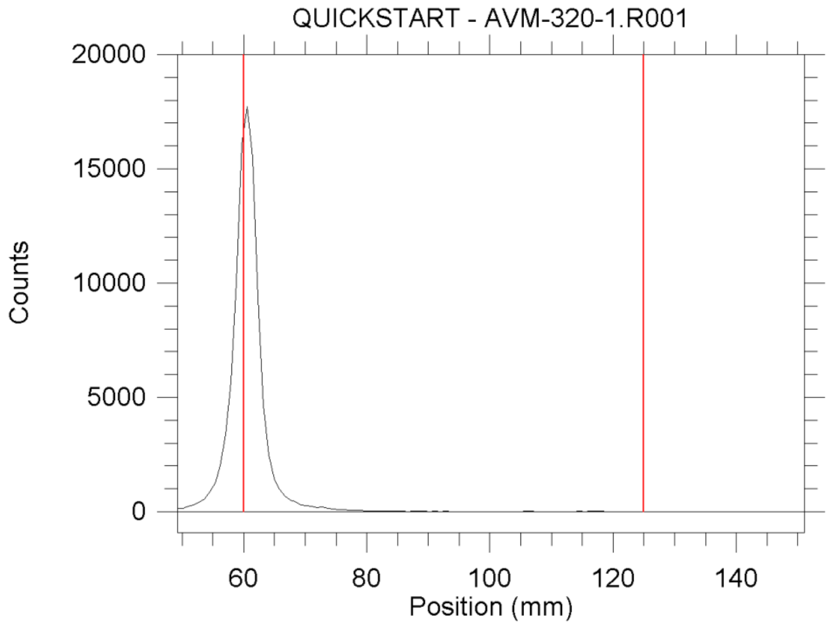

Figure 5.1.2B: Radio-TLC spectrum of the crude reaction mixture from 5.1.2

5.1.3

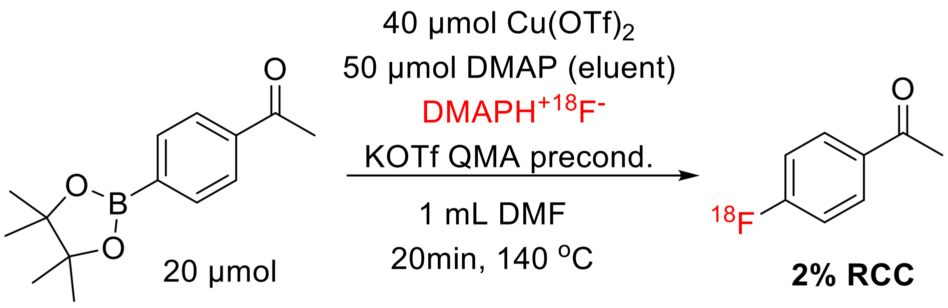

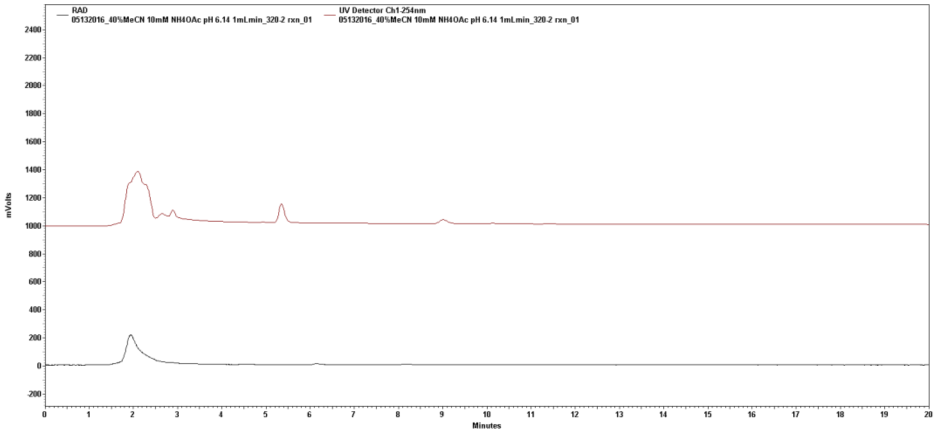

Figure 5.1.3A: HPLC spectra of crude reaction mixture from 5.1.3. RAD trace (black, bottom) and UV trace (256 nm, red, top). HPLC conditions A.

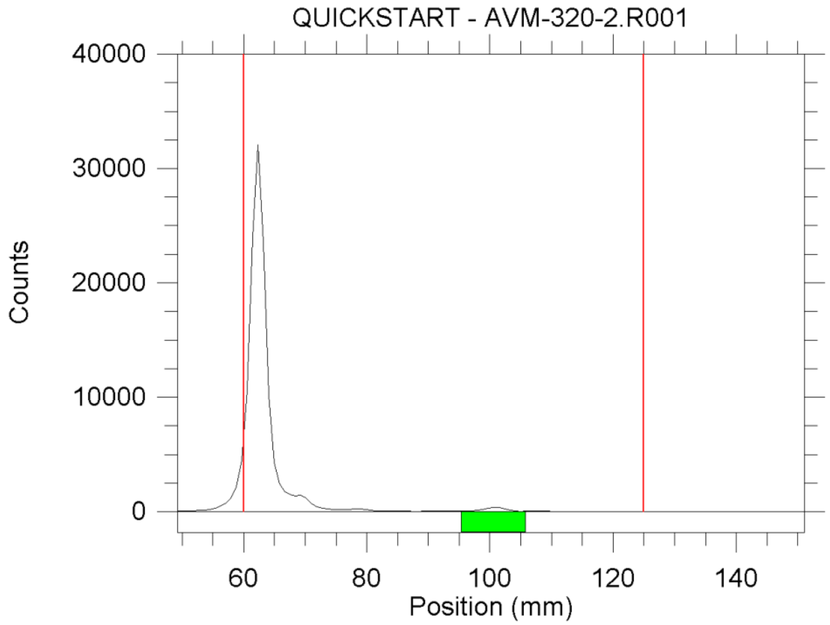

Figure 5.1.3B: Radio-TLC spectrum of the crude reaction mixture from 5.1.3.

5.1.4

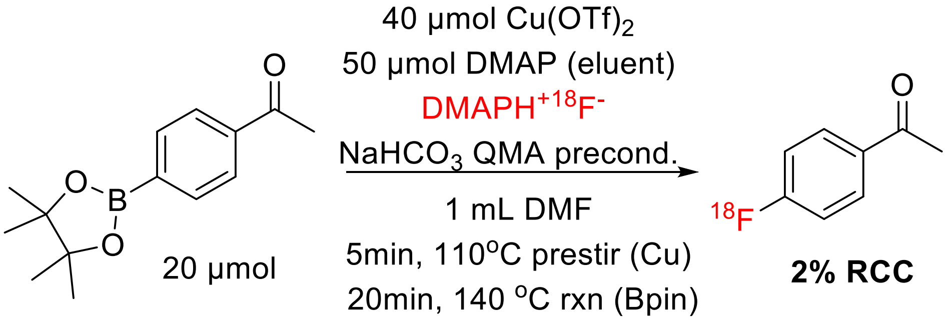

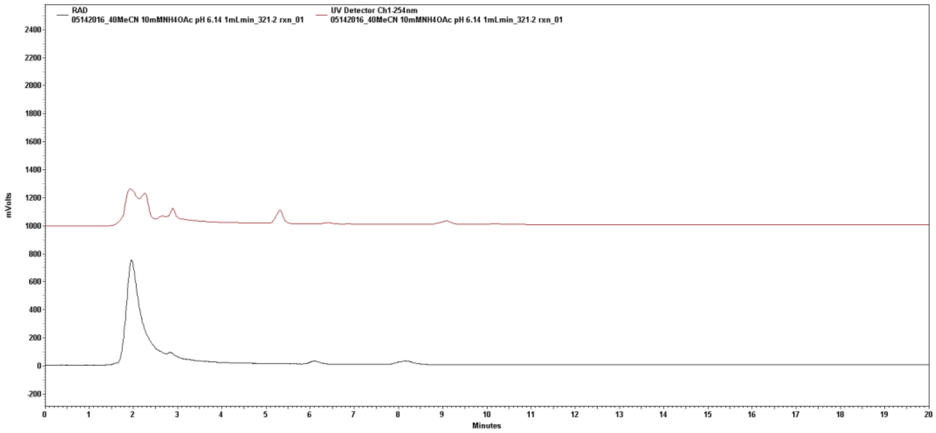

Figure 5.1.4A: HPLC spectra of crude reaction mixture from 5.1.4. RAD trace (black, bottom) and UV trace (256 nm, red, top). HPLC conditions A.

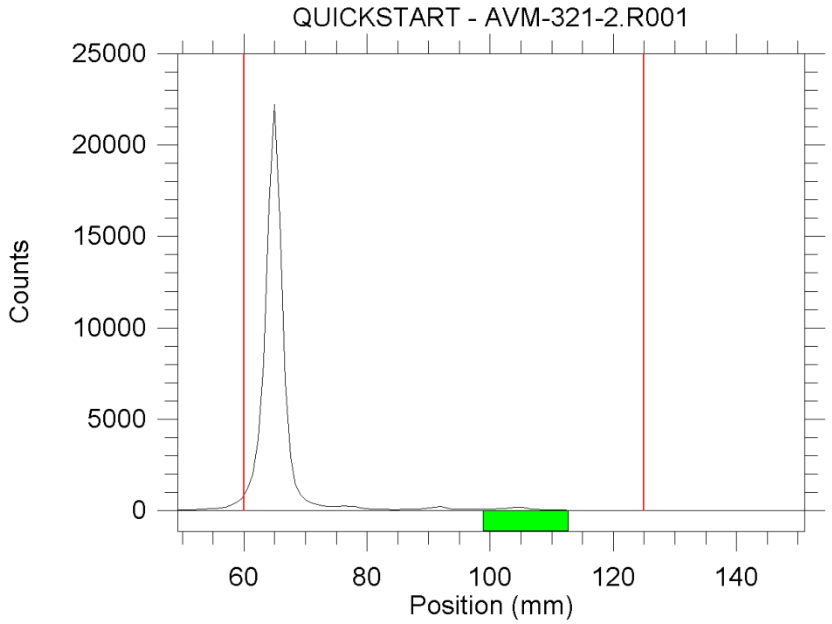

Figure 5.1.4B: Radio-TLC spectrum of the crude reaction mixture from 5.1.4

5.1.5

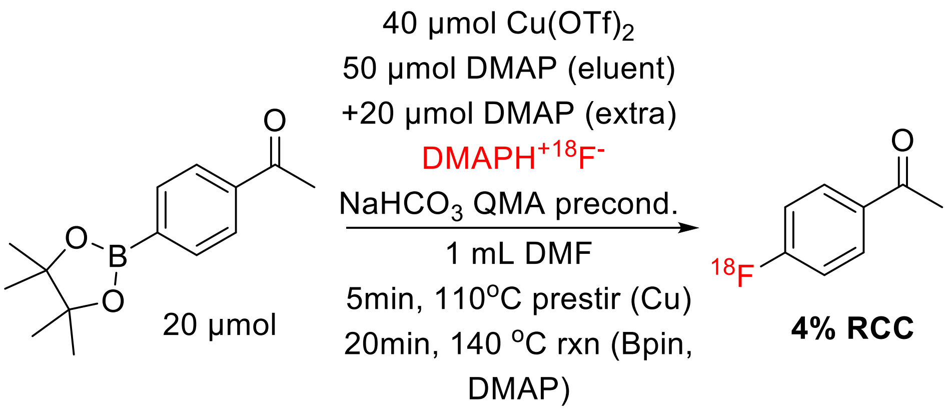

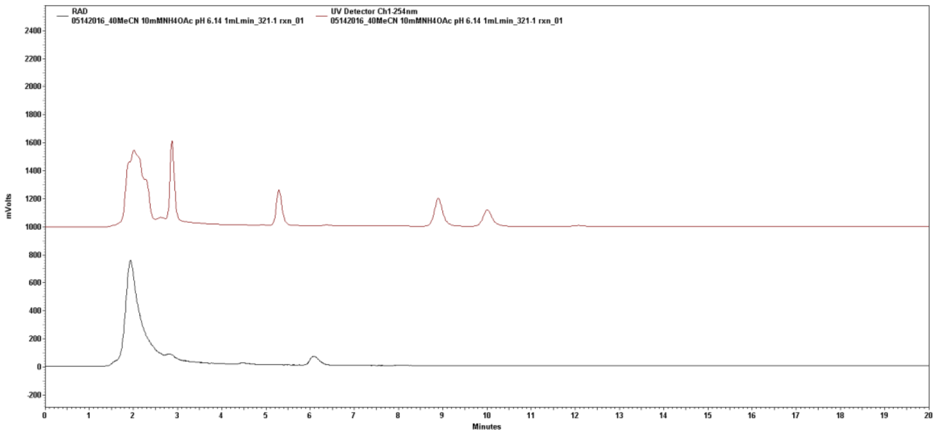

Figure 5.1.5A: HPLC spectra of crude reaction mixture from 5.1.5. RAD trace (black, bottom) and UV trace (256 nm, red, top). HPLC conditions A.

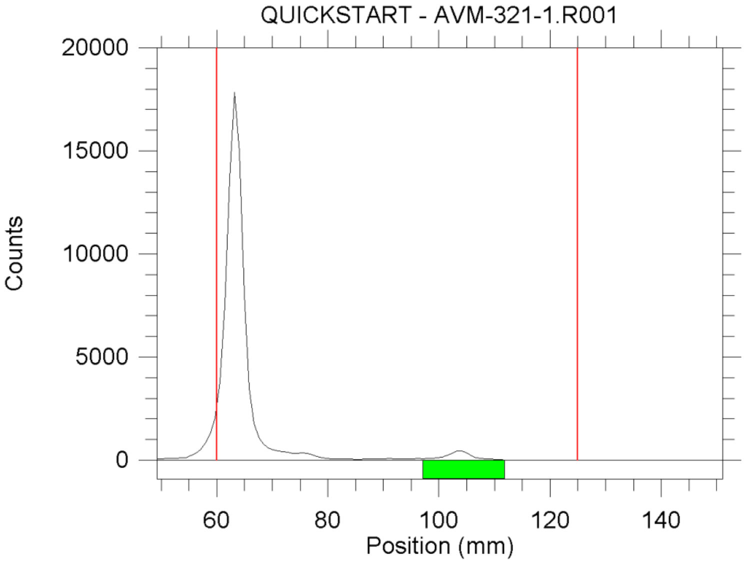

Figure 5.1.5B: Radio-TLC spectrum of the crude reaction mixture from 5.1.5.

5.1.6

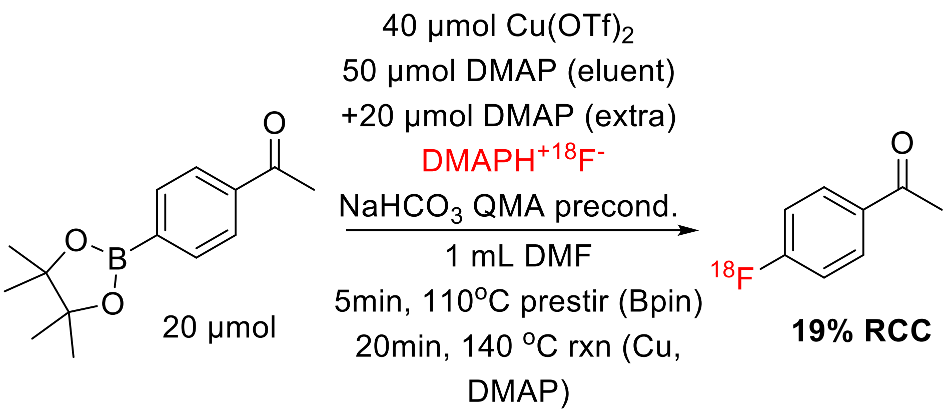

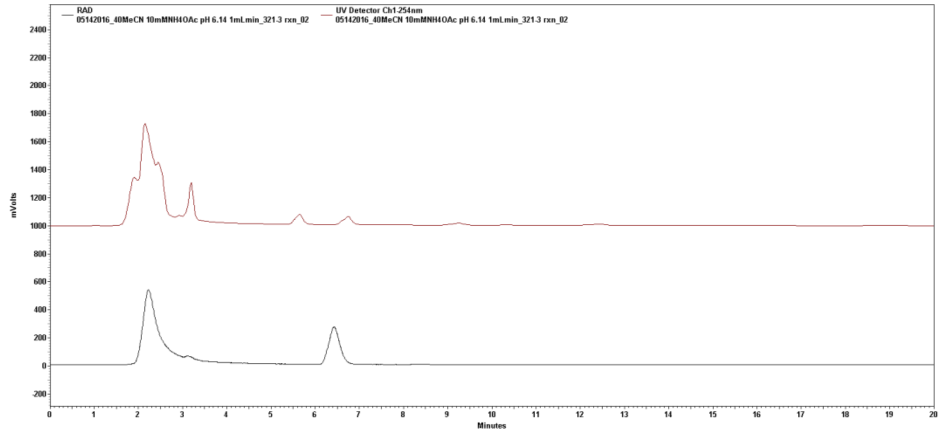

Figure 5.1.6A: HPLC spectra of crude reaction mixture from 5.1.6. RAD trace (black, bottom) and UV trace (256 nm, red, top). HPLC conditions A.

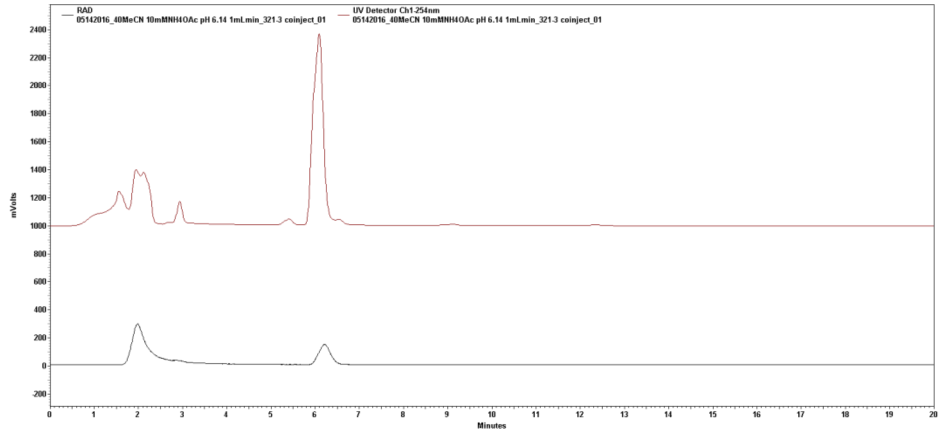

Figure 5.1.6B: HPLC spectra of crude reaction mixture from 5.1.6. spiked with 4-fluoroacetophenone reference standard. RAD trace (black, bottom) and UV trace (256 nm, red, top). HPLC conditions A.

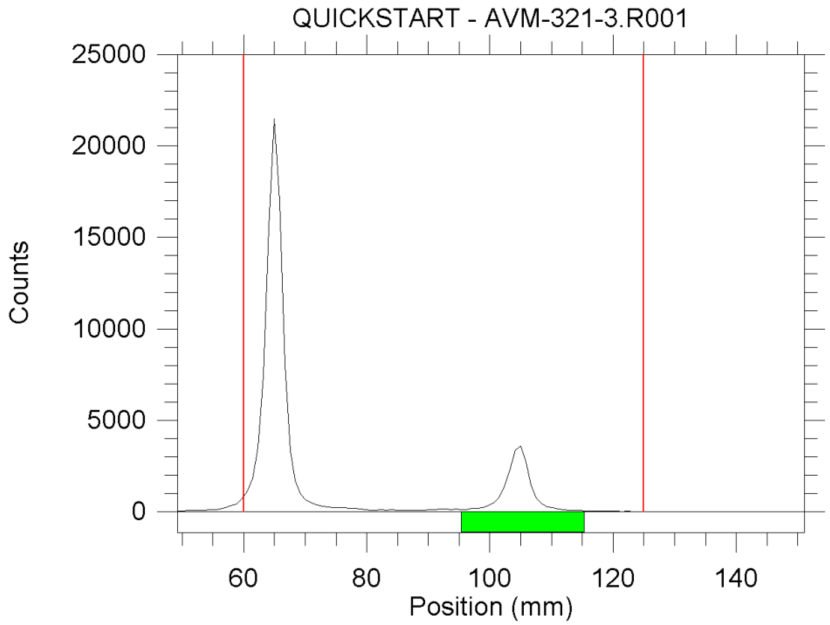

Figure 5.1.6C: Radio-TLC spectrum of the crude reaction mixture from 5.1.6.

5.1.7

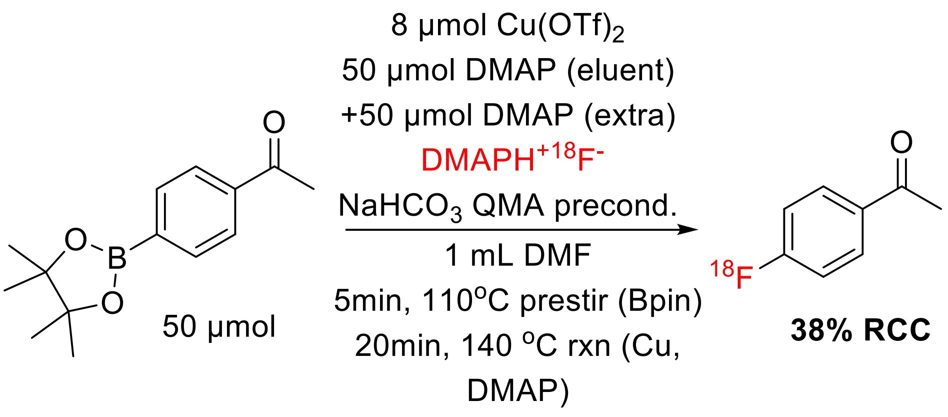

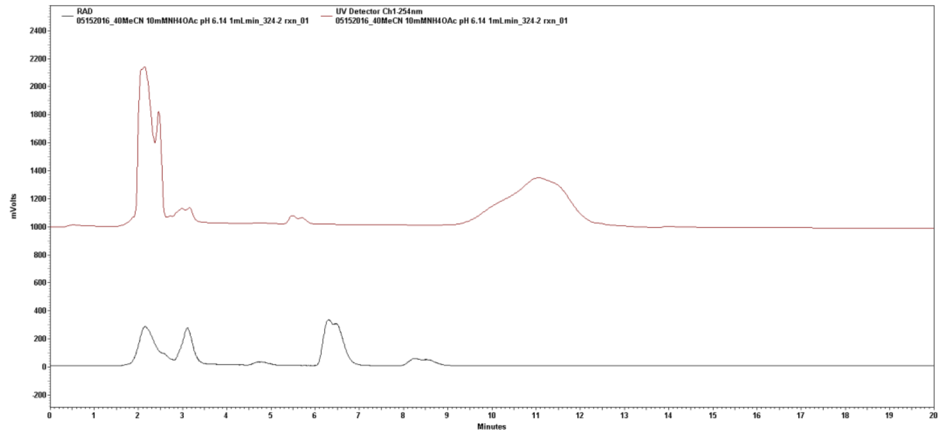

Figure 5.1.7A: HPLC spectra of crude reaction mixture from 5.1.7. RAD trace (black, bottom) and UV trace (256 nm, red, top). HPLC conditions A.

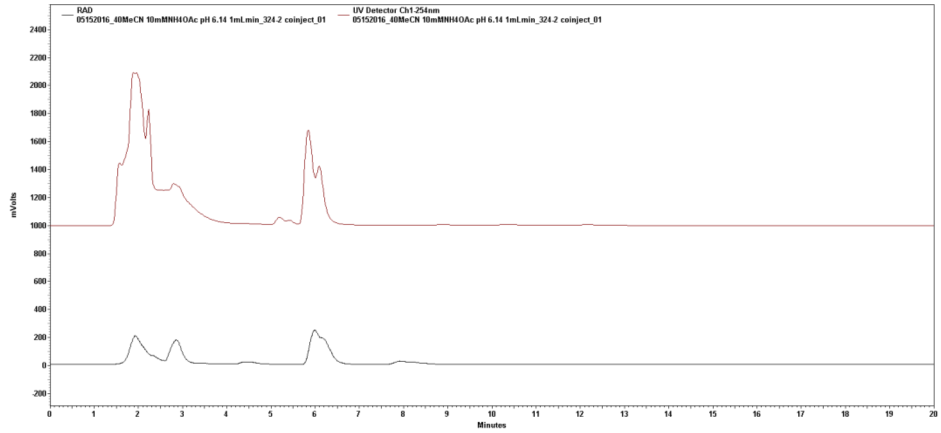

Figure 5.1.7B: HPLC spectra of crude reaction mixture from 5.1.7. spiked with 4-fluoroacetophenone reference standard. RAD trace (black, bottom) and UV trace (256 nm, red, top). HPLC conditions A.

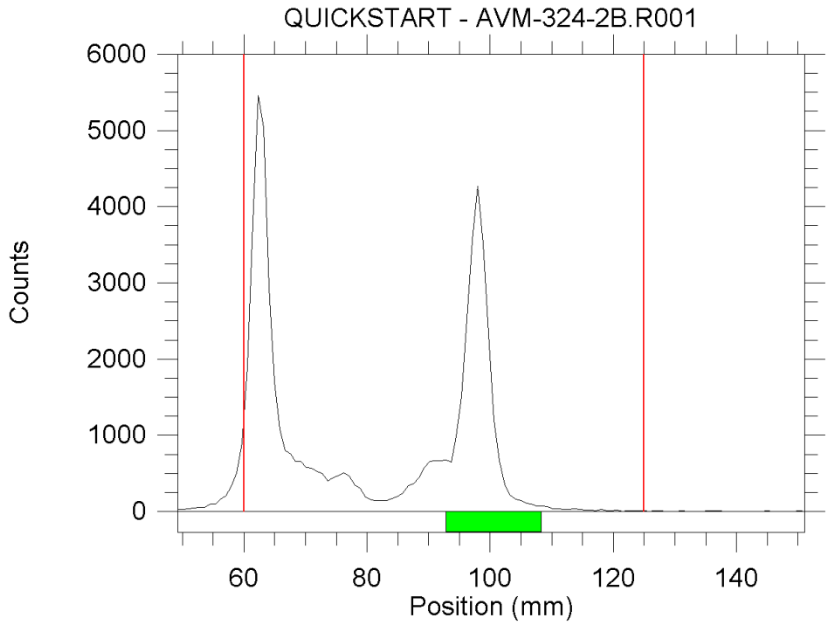

Figure 5.1.7C: Radio-TLC spectrum of the crude reaction mixture from 5.1.7.

5.1.8

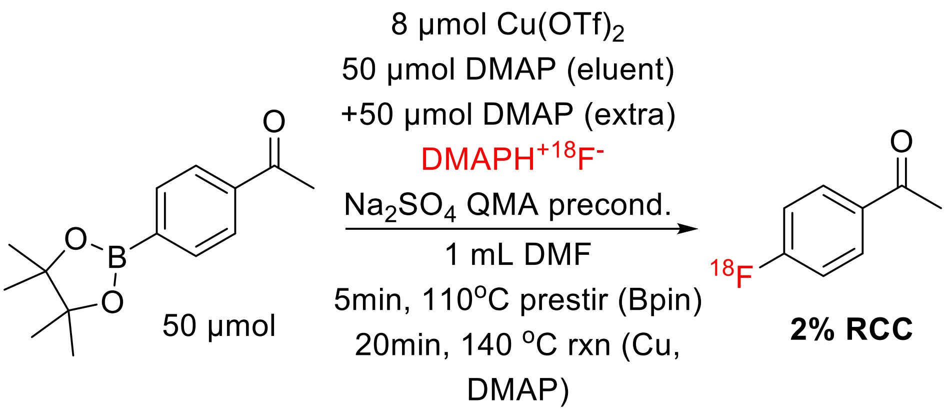

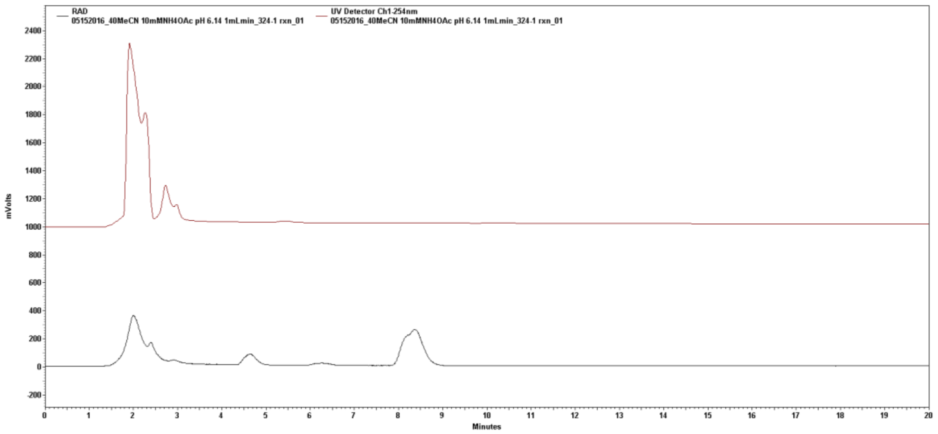

Figure 5.1.8A: HPLC spectra of crude reaction mixture from 5.1.8. RAD trace (black, bottom) and UV trace (256 nm, red, top). HPLC conditions A.

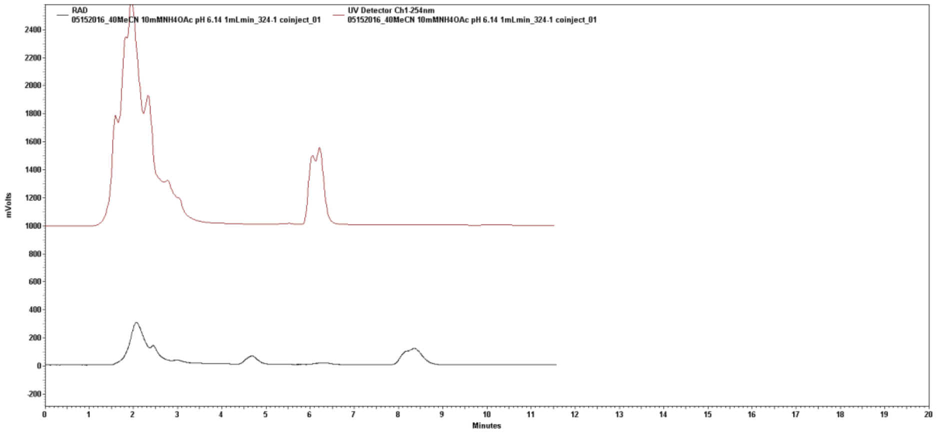

Figure 5.1.8B: HPLC spectra of crude reaction mixture from 5.1.8. spiked with 4-fluoroacetophenone reference standard. RAD trace (black, bottom) and UV trace (256 nm, red, top). HPLC conditions A.

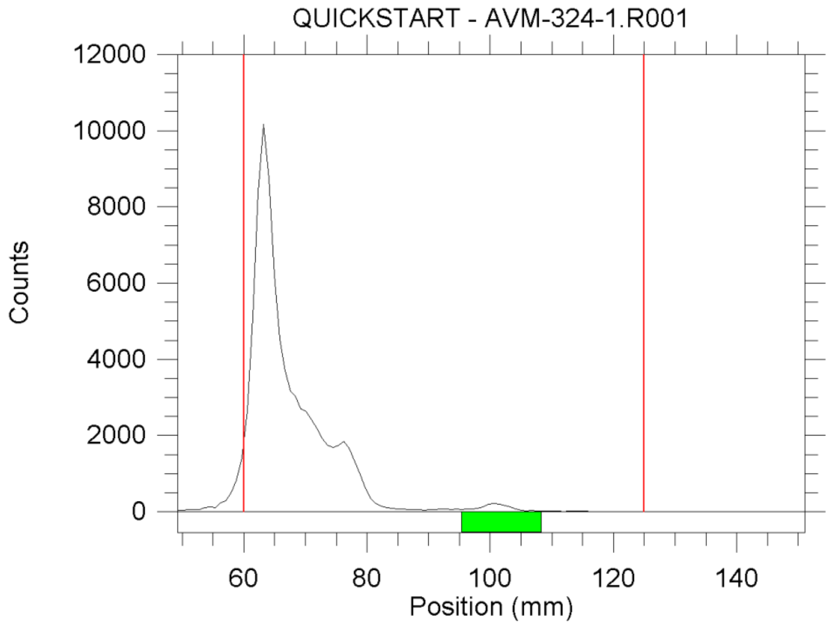

Figure 5.1.8C: Radio-TLC spectrum of the crude reaction mixture from 5.1.8.

5.1.9

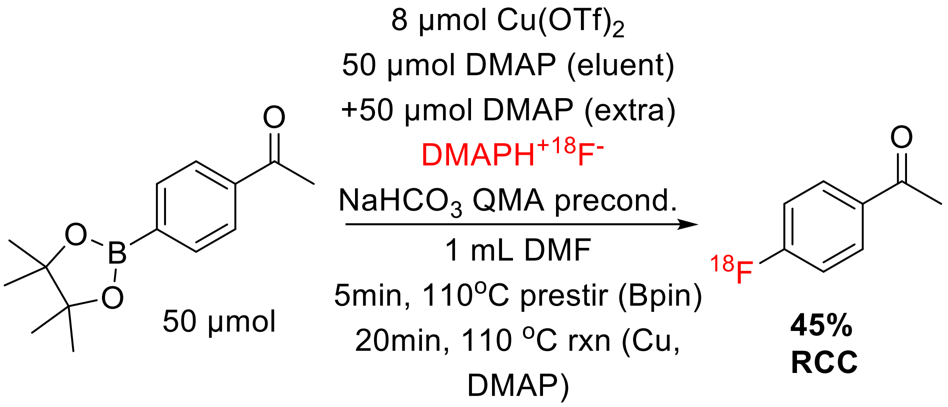

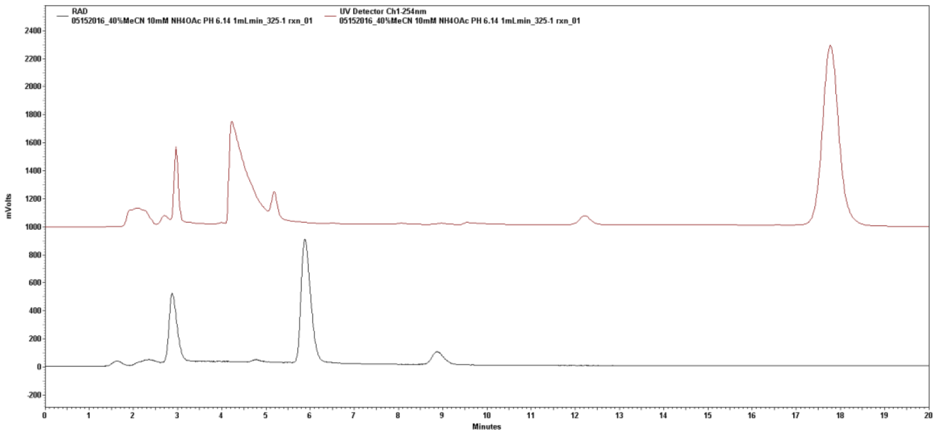

Figure 5.1.9A: HPLC spectra of crude reaction mixture from 5.1.9. RAD trace (black, bottom) and UV trace (256 nm, red, top). HPLC conditions B.

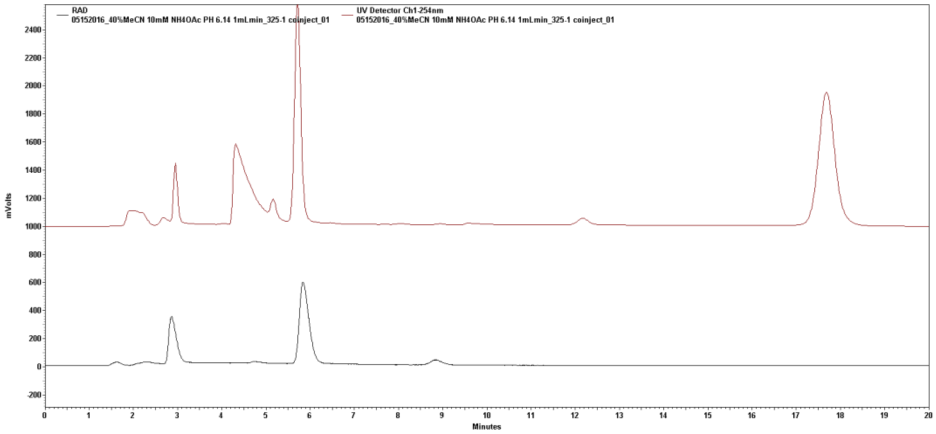

Figure 5.1.9B: HPLC spectra of crude reaction mixture from 5.1.9. spiked with 4-fluoroacetophenone reference standard. RAD trace (black, bottom) and UV trace (256 nm, red, top). HPLC conditions B.

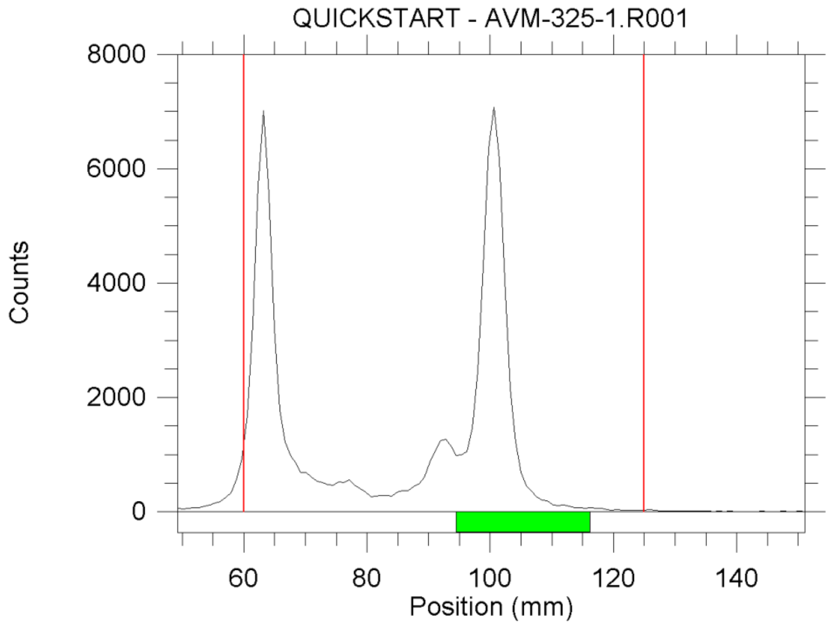

Figure 5.1.9C: Radio-TLC spectrum of the crude reaction mixture from 5.1.9.

5.1.10

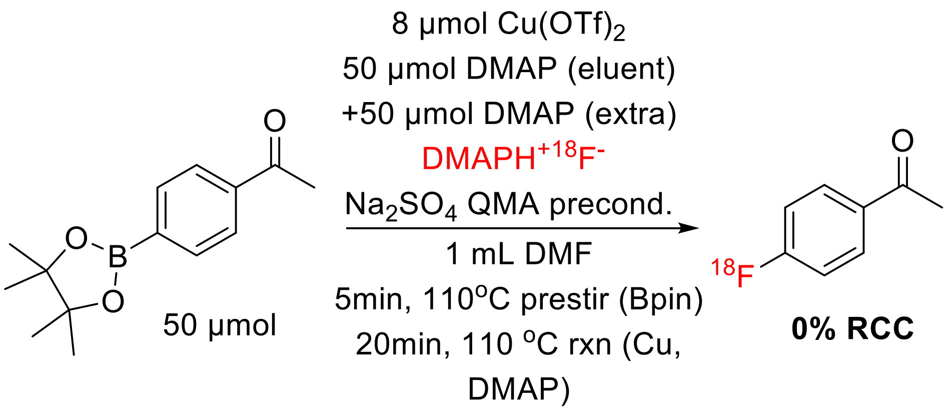

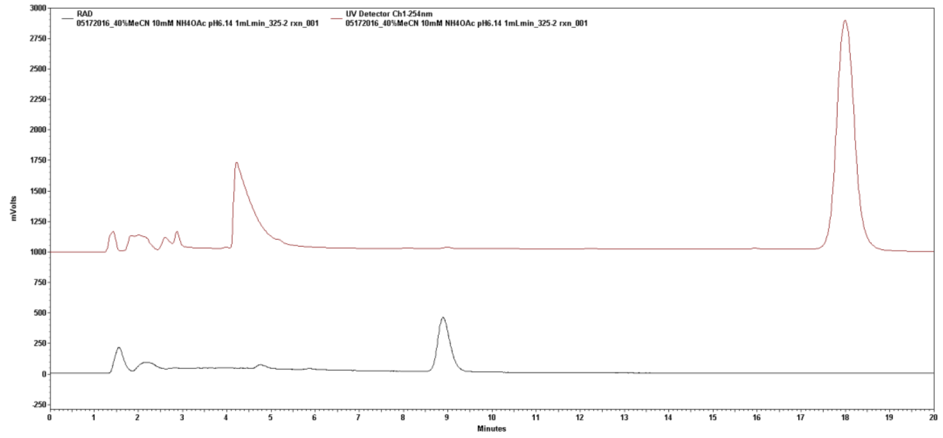

Figure 5.1.10A: HPLC spectra of crude reaction mixture from 5.1.10. RAD trace (black, bottom) and UV trace (256 nm, red, top). HPLC conditions B.

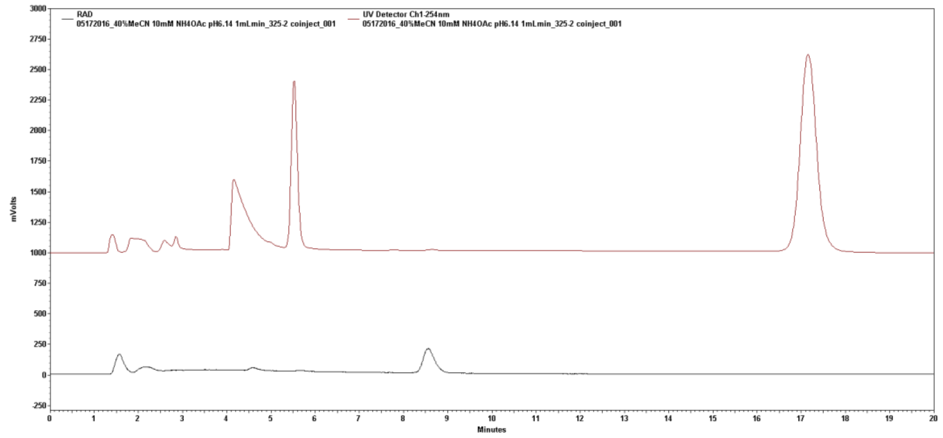

Figure 5.1.10B: HPLC spectra of crude reaction mixture from 5.1.10. spiked with 4-fluoroacetophenone reference standard. RAD trace (black, bottom) and UV trace (256 nm, red, top). HPLC conditions B.

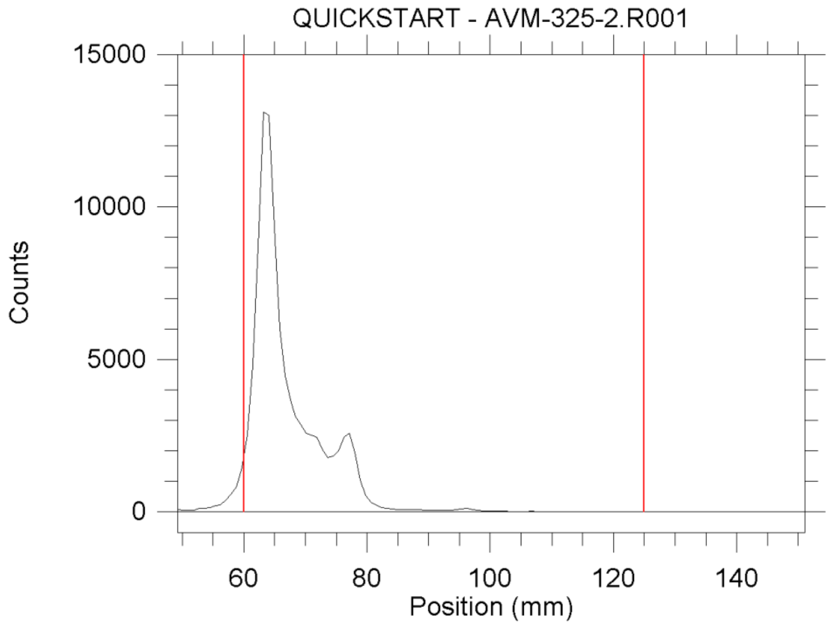

Figure 5.1.10C: Radio-TLC spectrum of the crude reaction mixture from 5.1.10.

5.2 Synthesis of [^{18}F]4-fluorophenacylbromide with DMAP elution

5.2.1

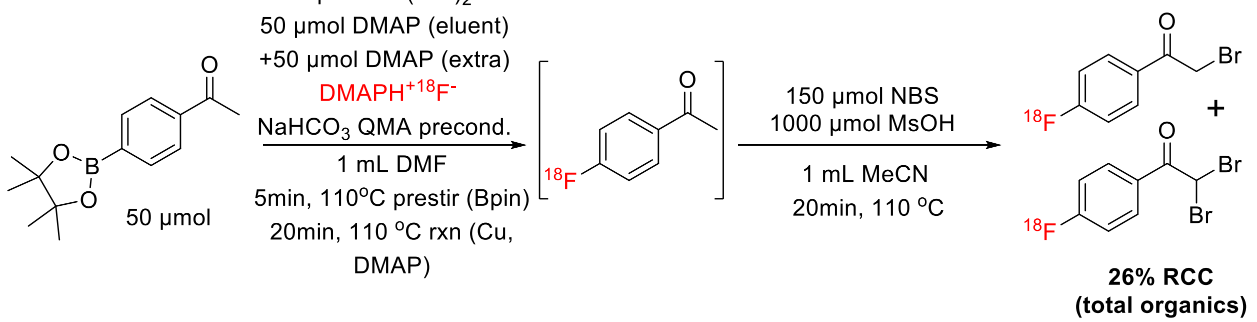

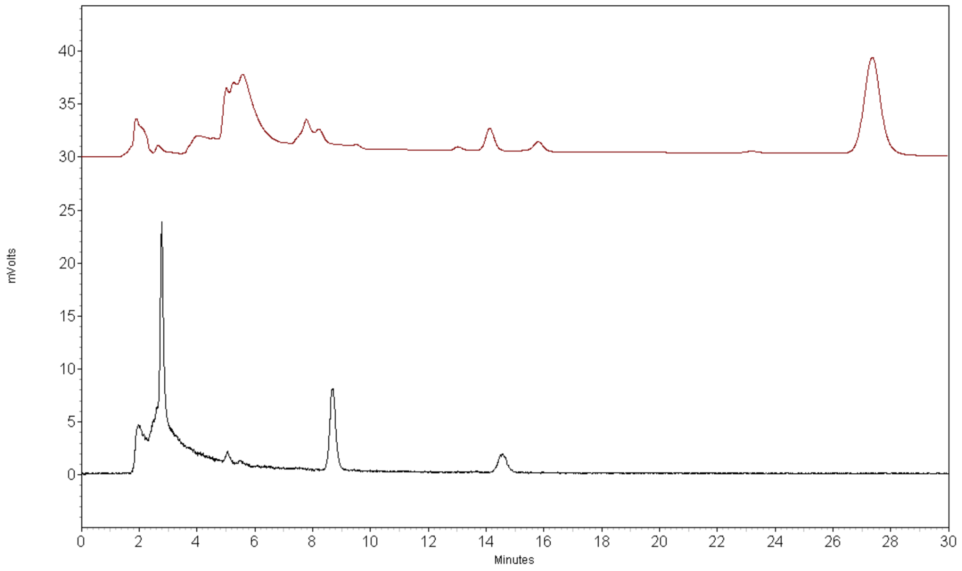

Figure 5.2.1A: HPLC spectra of crude reaction mixture from 5.2.1. RAD trace (black, bottom) and UV trace (256 nm, red, top). HPLC conditions B.

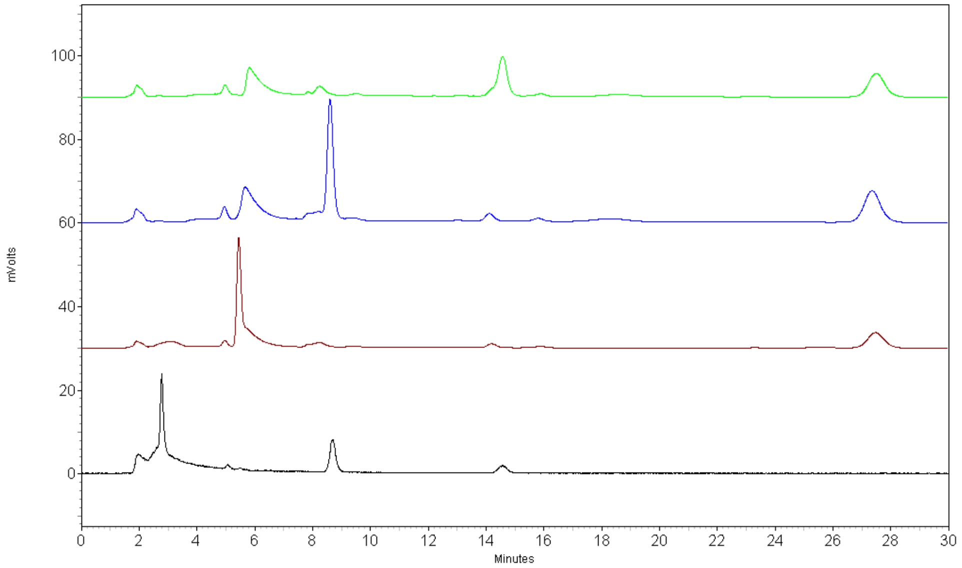

Figure 5.2.1B: HPLC spectra of crude reaction mixture from 5.2.1 spiked with reference standards. RAD trace (black, bottom) and UV trace (256 nm) with FAP (red), FPB (blue), FPDB (green). HPLC conditions B.

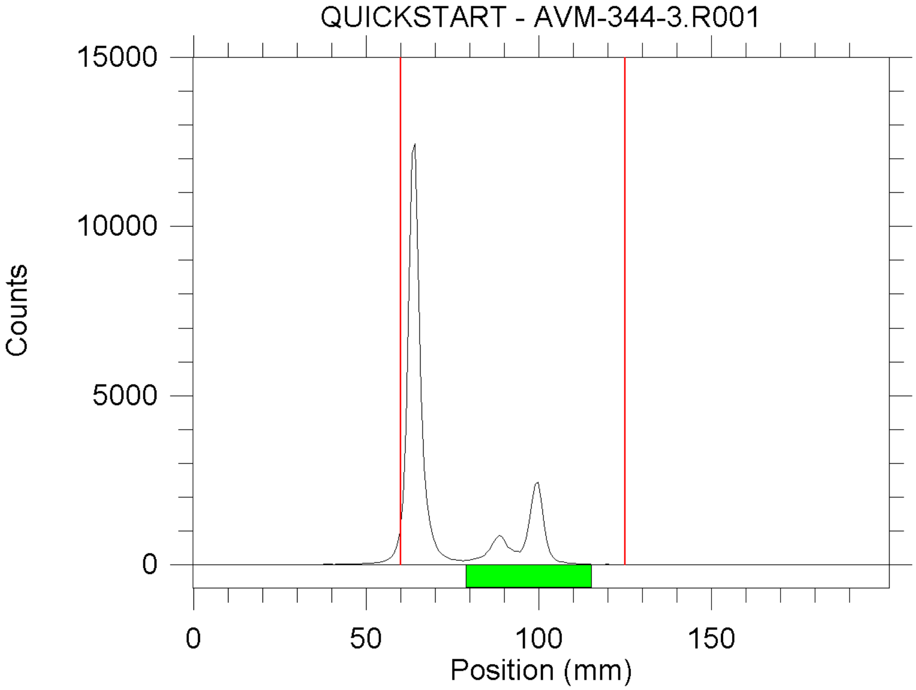

Figure 5.2.1C: Radio-TLC spectrum of the crude reaction mixture from 5.2.1.

5.2.2

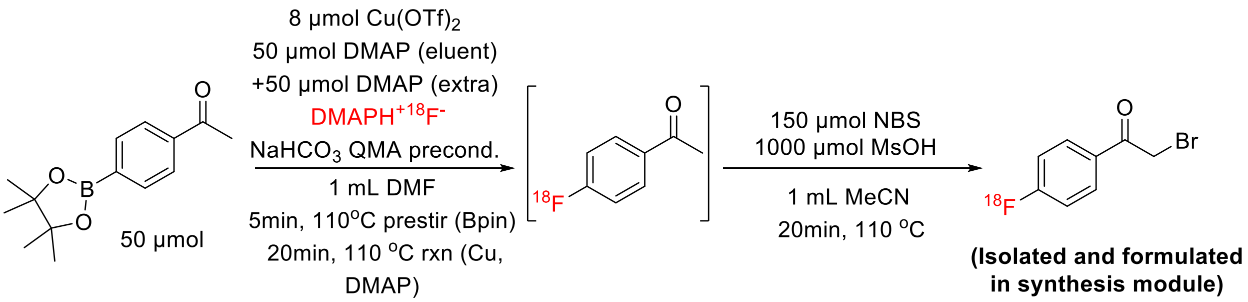

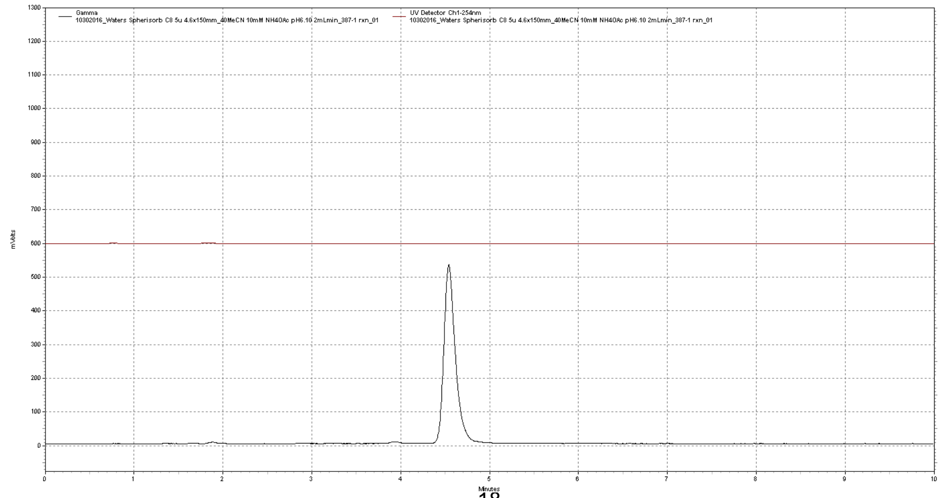

Figure 5.2.2A: HPLC spectra of formulated [^{18}F]FPB from 5.2.2. RAD trace (black, bottom) and UV trace (256 nm, red, top). HPLC conditions D.

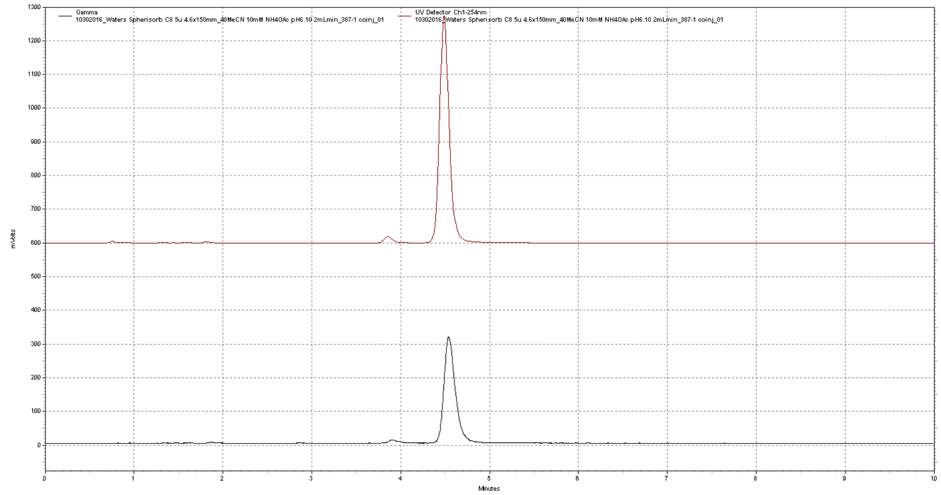

Figure 5.2.2B: HPLC spectra of crude reaction mixture from 5.2.2 spiked with FPB reference standard. RAD trace (black, bottom) and UV trace (256 nm). HPLC conditions D.

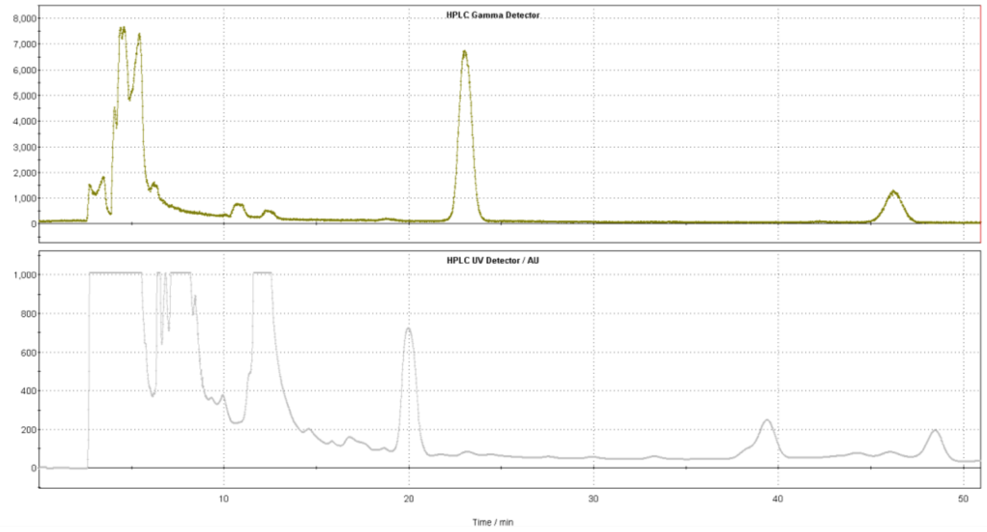

Figure 5.2.2C: Semipreparative HPLC spectrum during purification of the crude reaction mixture from 5.2.2.

5.3 Synthesis of [^{18}F]-4-fluoroacetophenone with $Cu(OTf)_2$ elution

5.3.1

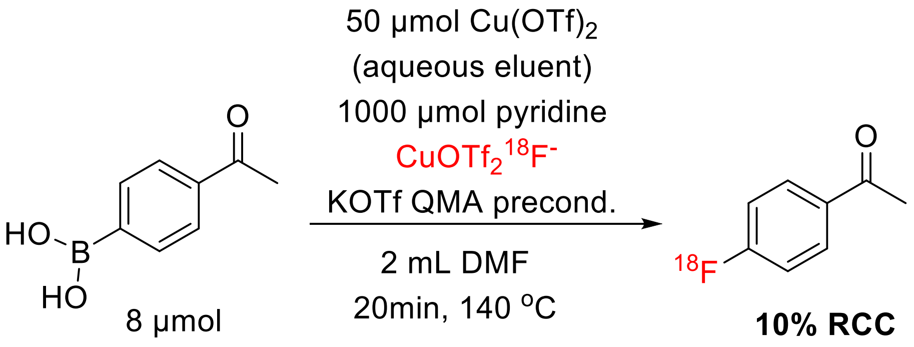

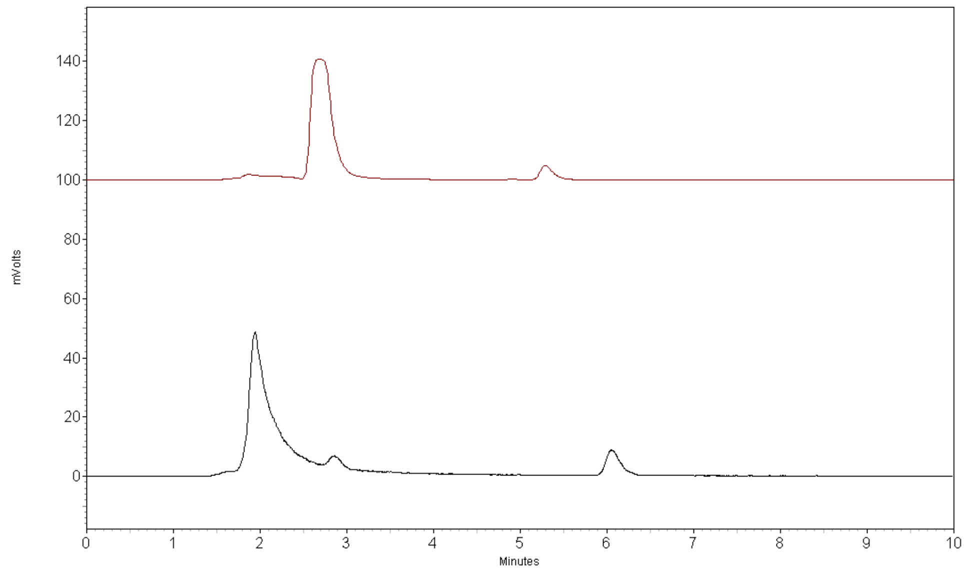

Figure 5.3.1A: HPLC spectra of crude reaction mixture from 5.3.1 RAD trace (black, bottom) and UV trace (256 nm, red, top). HPLC conditions A.

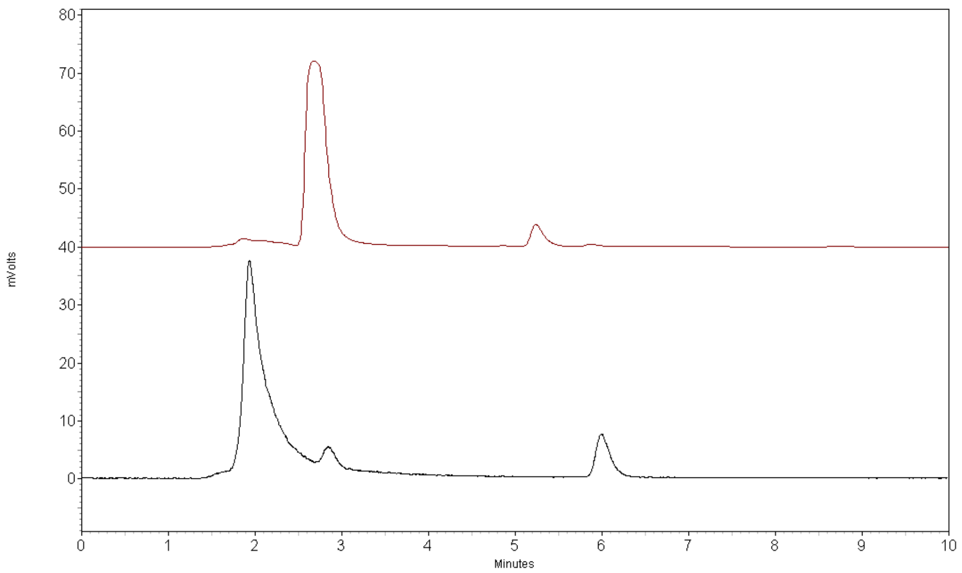

Figure 5.3.1B: HPLC spectra of crude reaction mixture from 5.3.1spiked with 4-fluoroacetophenone reference standard (note: standard 254nm signal is unusually small). RAD trace (black, bottom) and UV trace (256 nm, red, top). HPLC conditions A.

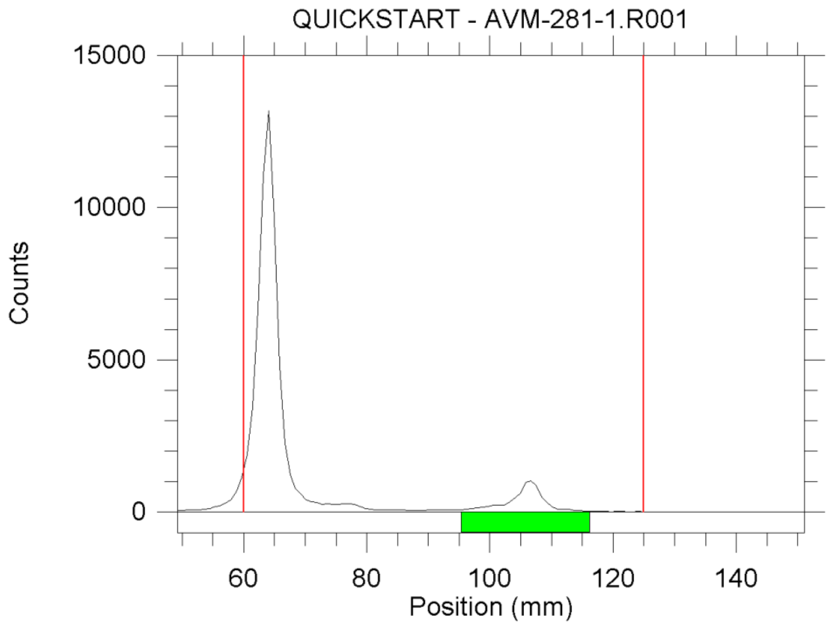

Figure 5.3.1C: Radio-TLC spectrum of the crude reaction mixture from 5.3.1

5.3.2

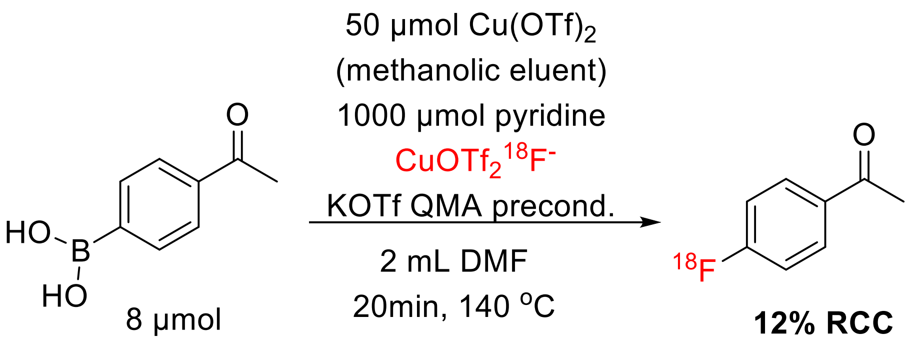

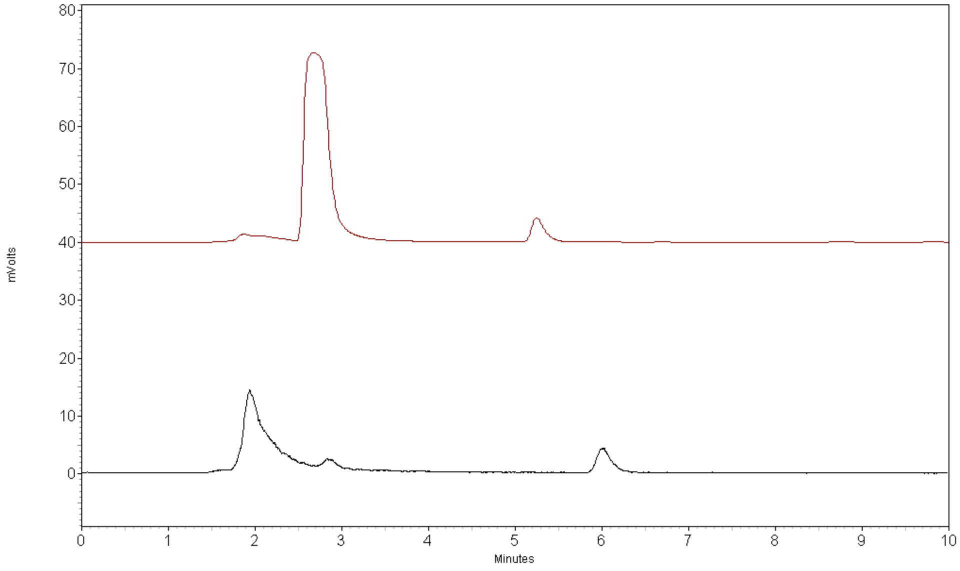

Figure 5.3.2A: HPLC spectra of crude reaction mixture from 5.3.2 RAD trace (black, bottom) and UV trace (256 nm, red, top). HPLC conditions A.

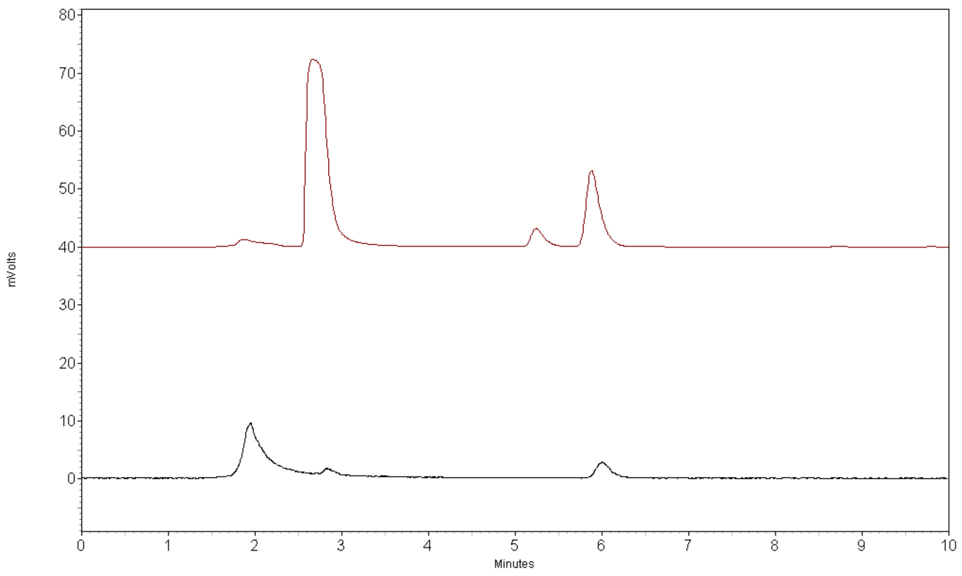

Figure 5.3.2B: HPLC spectra of crude reaction mixture from 5.3.2 spiked with 4-fluoroacetophenone reference standard. RAD trace (black, bottom) and UV trace (256 nm, red, top). HPLC conditions A.

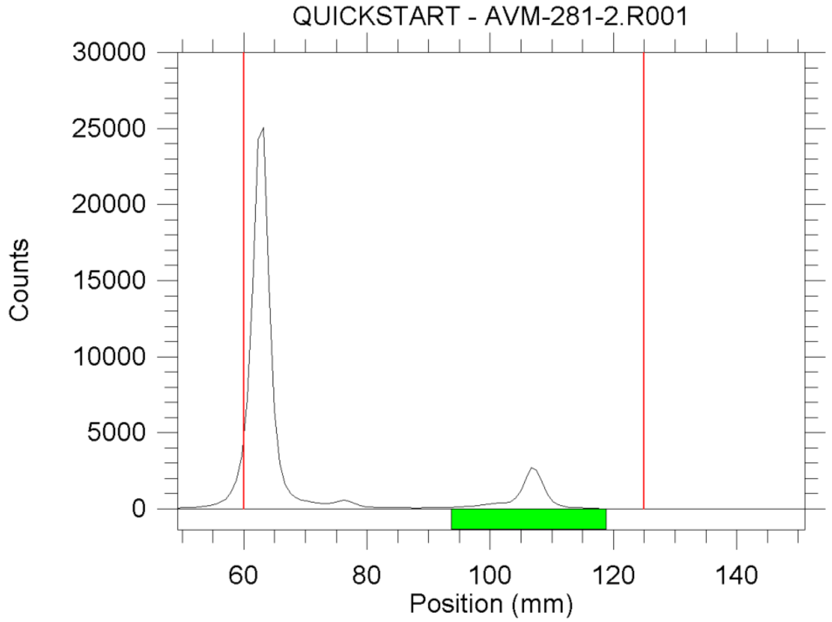

Figure 5.3.2C: Radio-TLC spectrum of the crude reaction mixture from 5.3.2

6. Substrate Scope Spectroscopic Data

6.1 4-[^{18}F]Fluoroacetophenone (2)

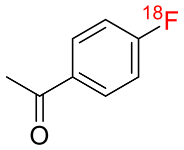

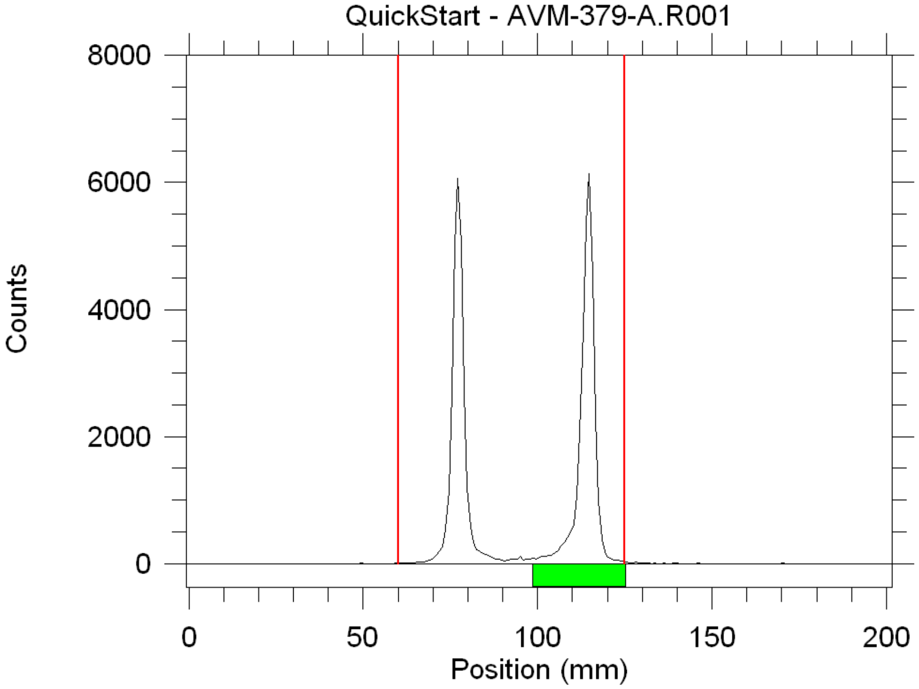

Figure 6.1A: 4-[^{18}F]Fluoroacetophenone (**2**) Radio-TLC spectrum

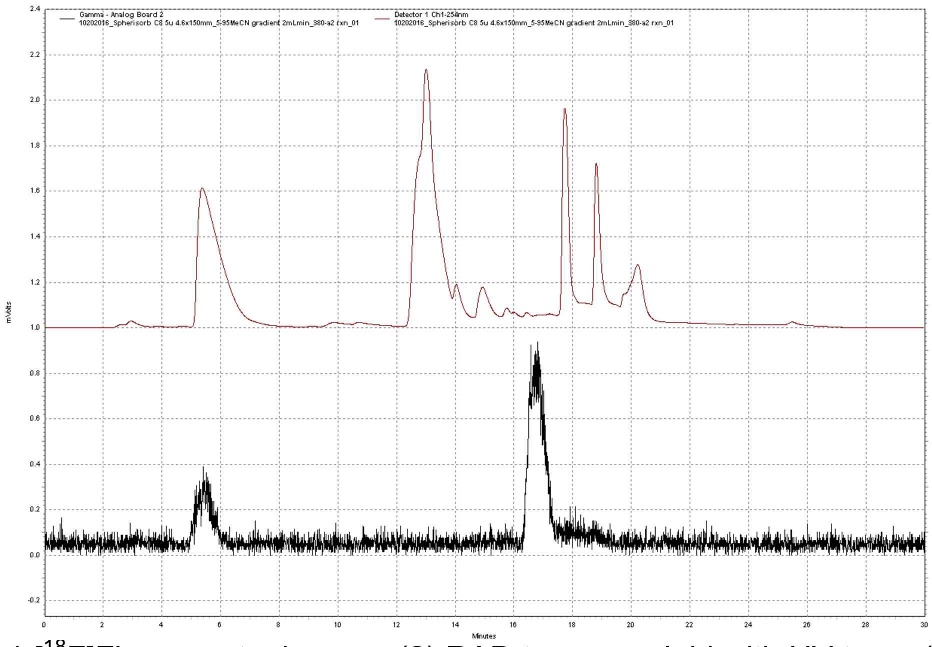

Figure 6.1B: 4-[^{18}F]Fluoroacetophenone (**2**) RAD trace overlaid with UV trace (256 nm), HPLC Conditions E

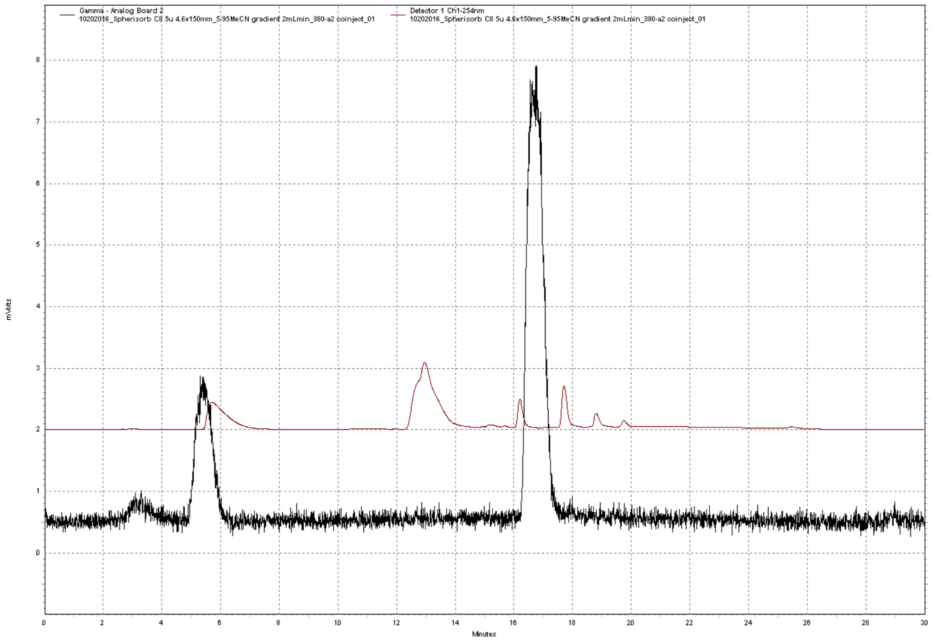

Figure 6.1C: 4-[^{18}F]Fluoroacetophenone (**2**) RAD trace overlaid with UV trace (256 nm) spiked with 4-fluoroacetophenone reference standard, HPLC Conditions E

6.2 2-[^{18}F]Fluoromethylbenzoate (4)

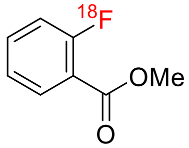

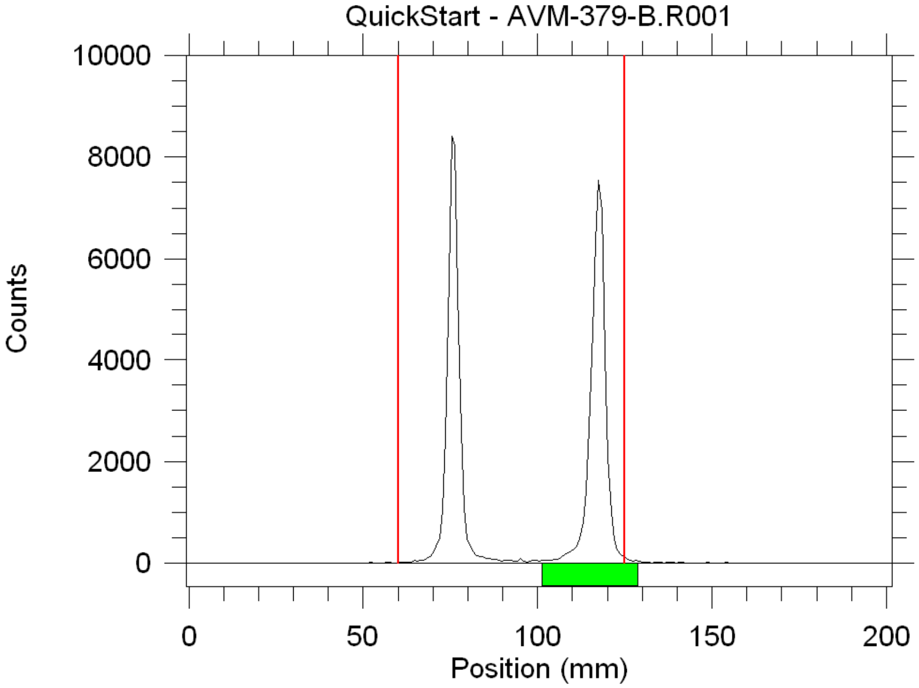

Figure 6.2A: 2-[^{18}F]fluoromethylbenzoate (**4**) Radio-TLC spectrum

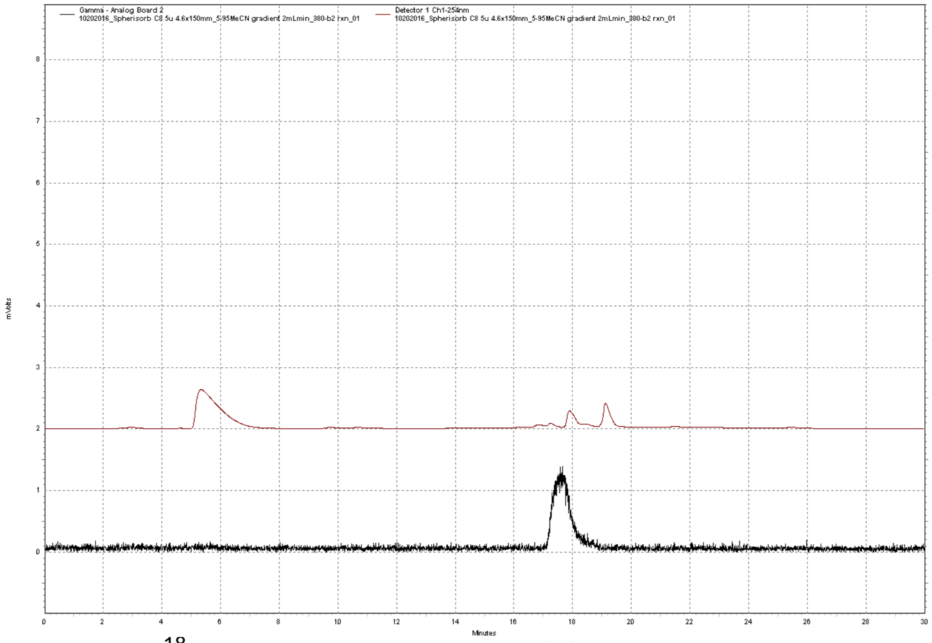

Figure 6.2B: 2-[^{18}F]Fluoromethylbenzoate (**4**) RAD trace overlaid with UV trace (256 nm), HPLC Conditions E

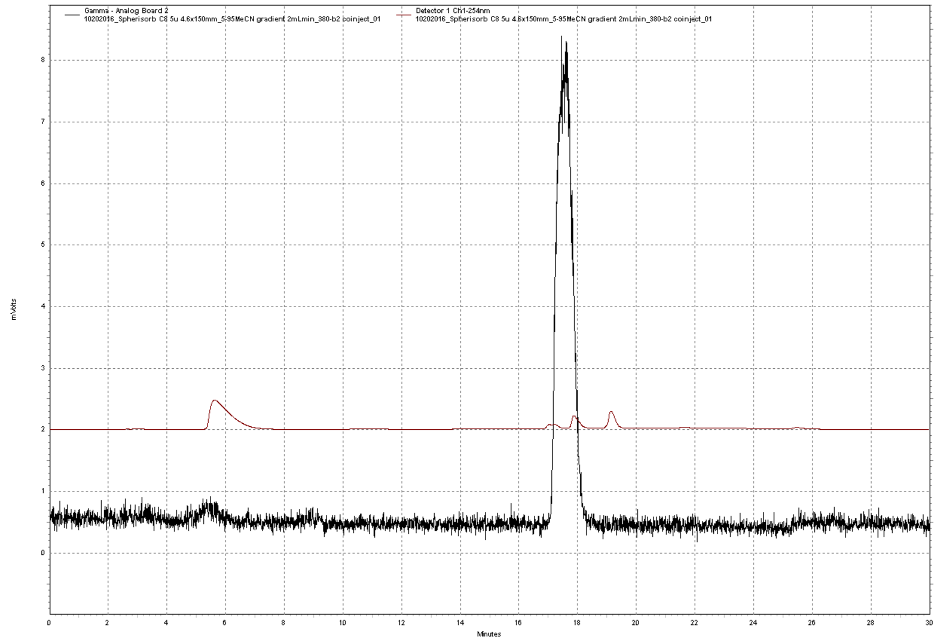

Figure 6.2C: 2-[^{18}F]Fluoromethylbenzoate (**4**) RAD trace overlaid with UV trace (256 nm) spiked with 2-fluoromethylbenzoate reference standard, HPLC Conditions E.

[**Note:** this compound was very poorly visible in the 200-300 nm range normally utilized for HPLC UV detection and can be seen as an increase in the size of the UV peak immediately prior to the RAD product peak].

6.3. 4-[^{18}F]Fluorobenzonitrile (5)

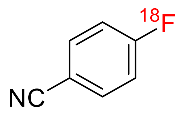

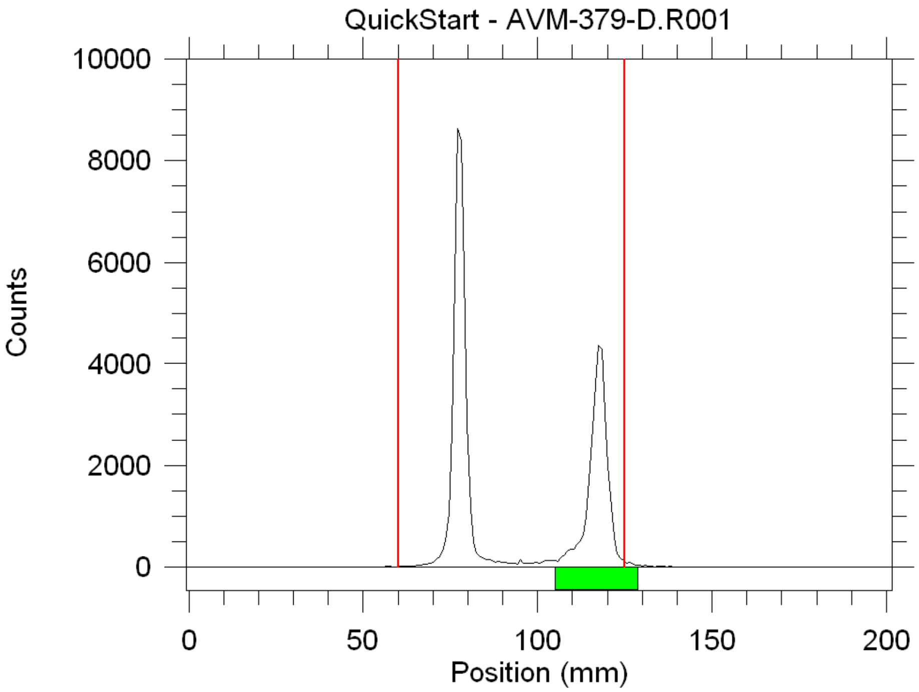

Figure 6.3A: 4-[^{18}F]Fluorobenzonitrile **(5)** Radio-TLC spectrum

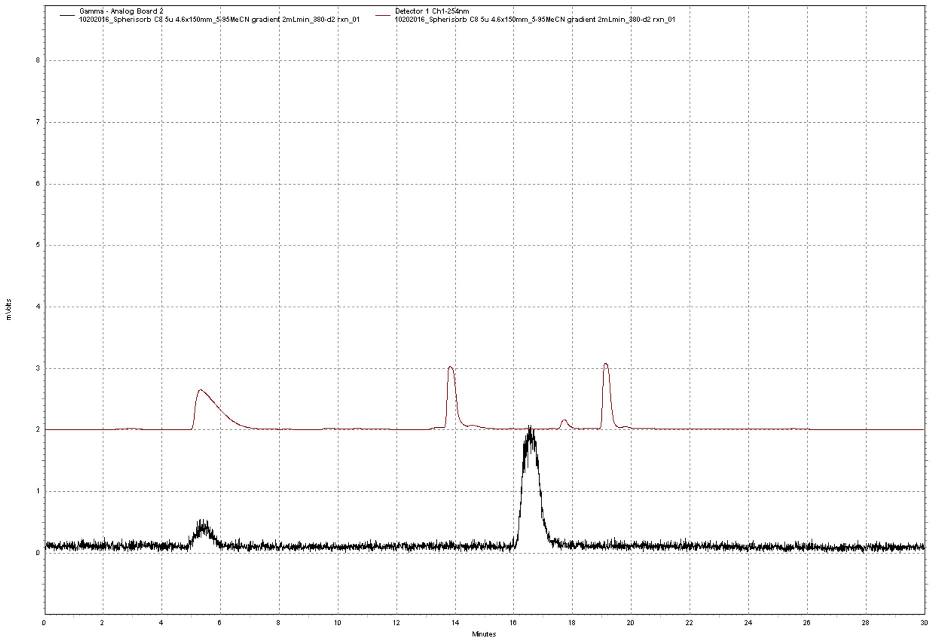

Figure 6.3B: 4-[^{18}F]Fluorobenzonitrile (**5**) RAD trace overlaid with UV trace (256 nm), HPLC Conditions E

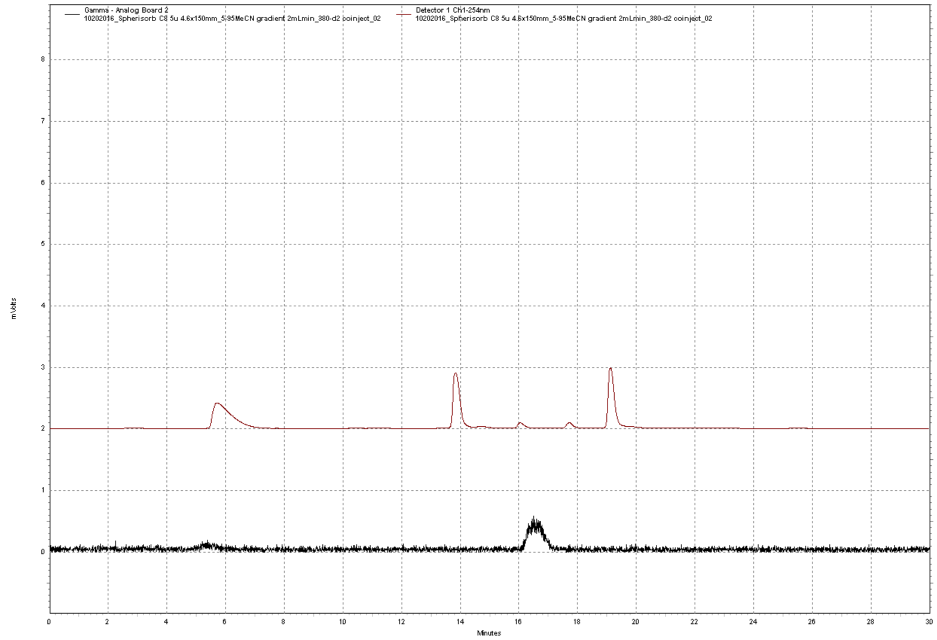

Figure 6.3C: 4-[^{18}F]Fluorobenzonitrile (**5**) RAD trace overlaid with UV trace (256 nm) spiked with 4-fluorobenzonitrile reference standard, HPLC Conditions E.

6.4 4-[^{18}F]Fluoronitrobenzene (6)

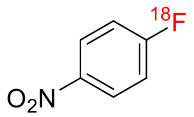

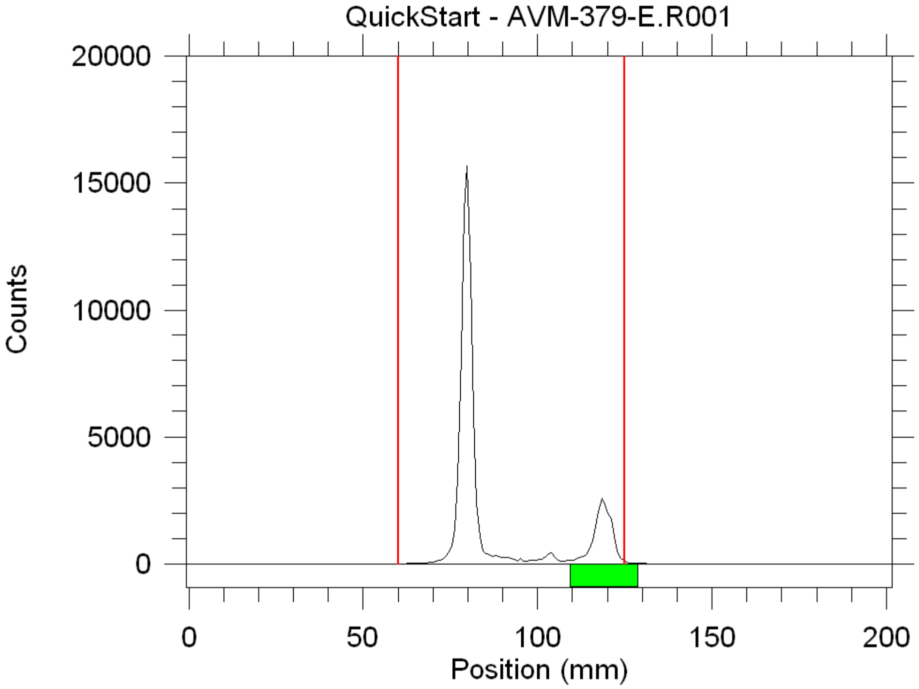

Figure 6.4A: 4-[^{18}F]Fluoronitrobenzene (**6**) Radio-TLC spectrum

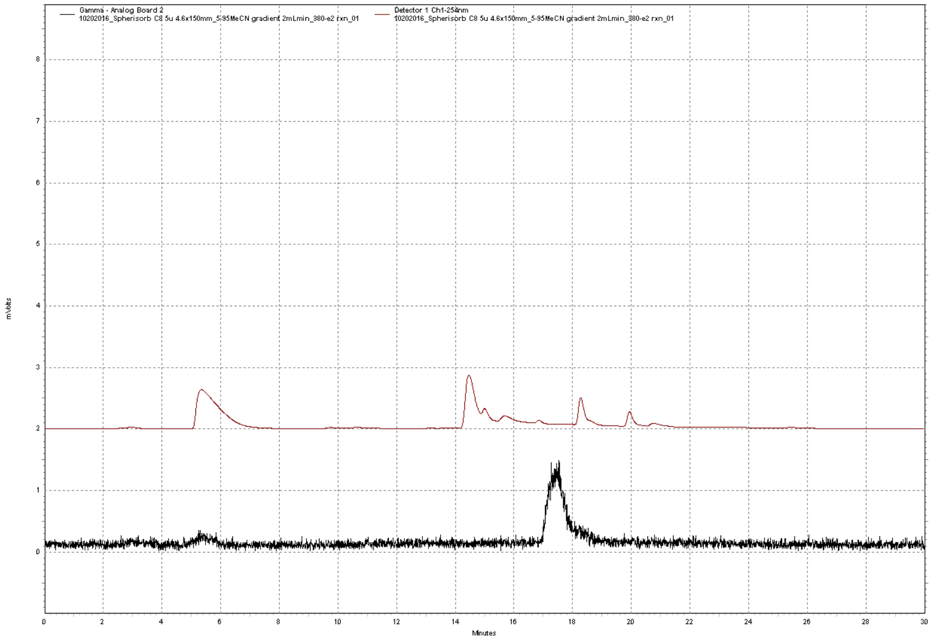

Figure 6.4B: 4-[^{18}F]Fluoronitrobenzene (**6**) RAD trace overlaid with UV trace (256 nm), HPLC Conditions E

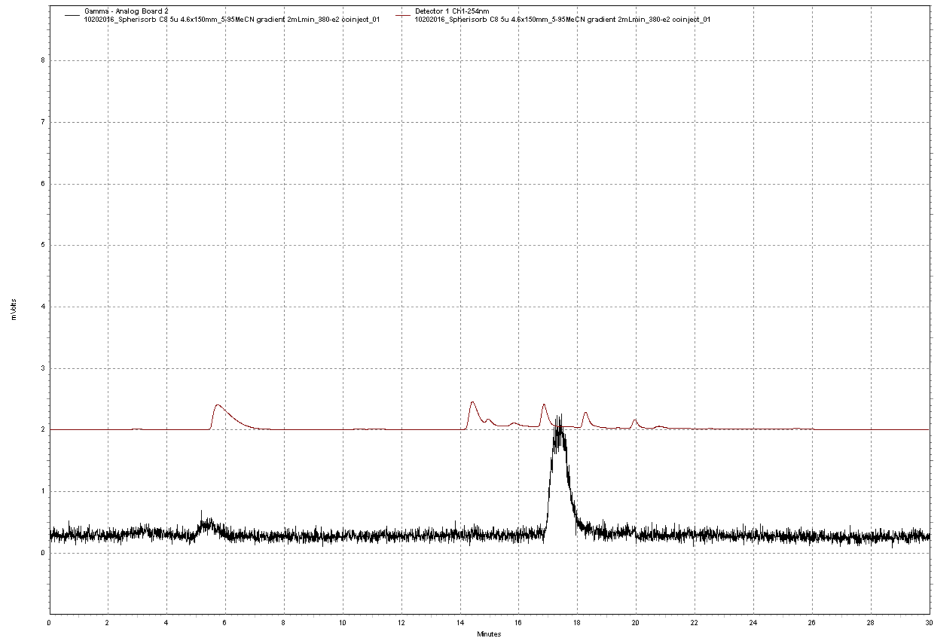

Figure 6.4C: 4-[^{18}F]Fluoronitrobenzene (**6**) RAD trace overlaid with UV trace (256 nm) spiked with 4-fluoronitrobenzene reference standard, HPLC Conditions E.

6.5 4-[^{18}F]-2,4-Difluoroaniline (7)

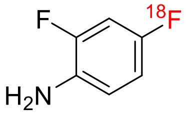

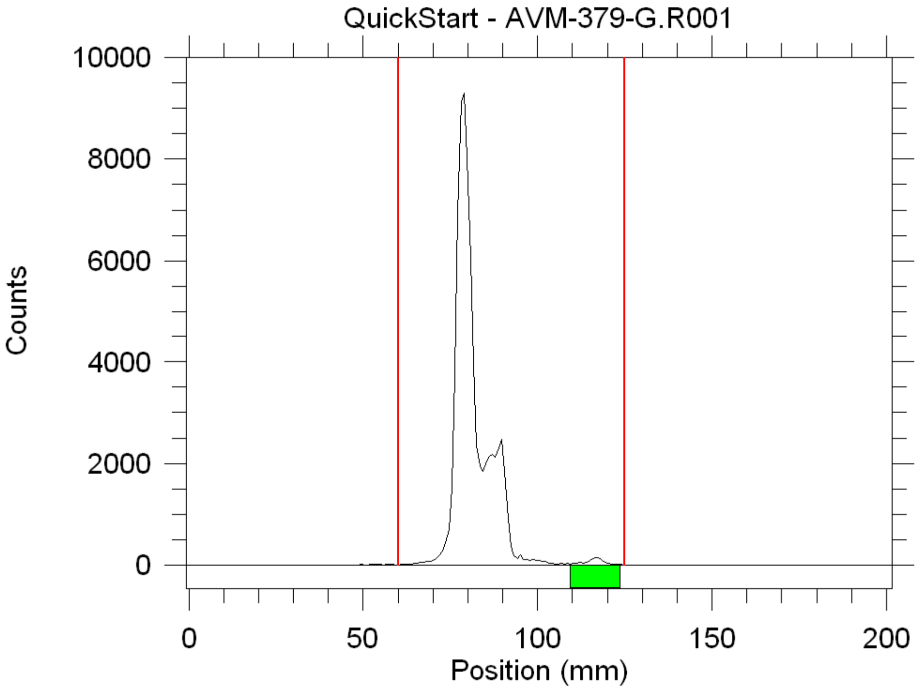

Figure 6.5A: 4-[^{18}F]-2,4-Difluoroaniline (**7**) Radio-TLC spectrum

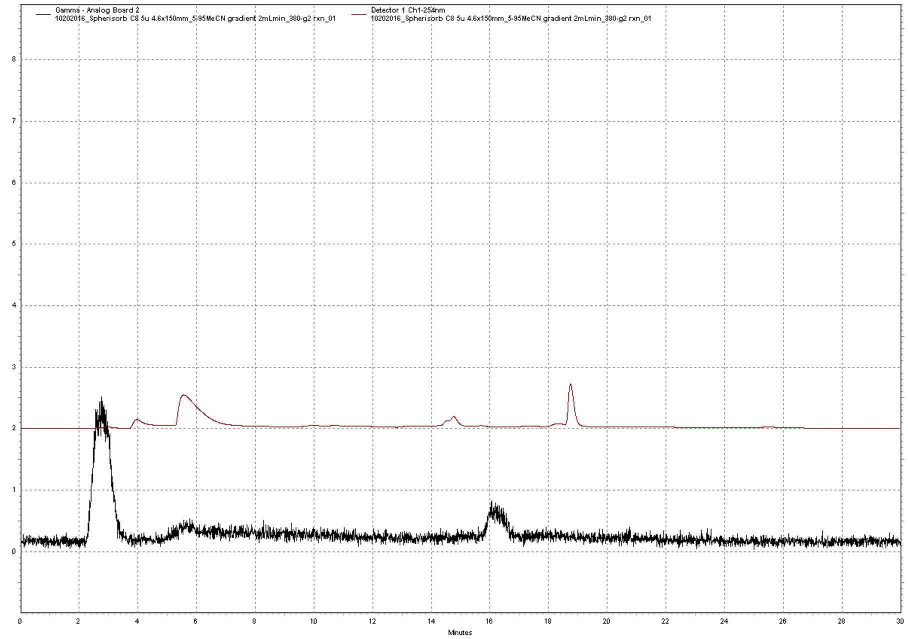

Figure 6.5B: 4-[^{18}F]-2,4-difluoroaniline (**7**) RAD trace overlaid with UV trace (256 nm), HPLC Conditions E

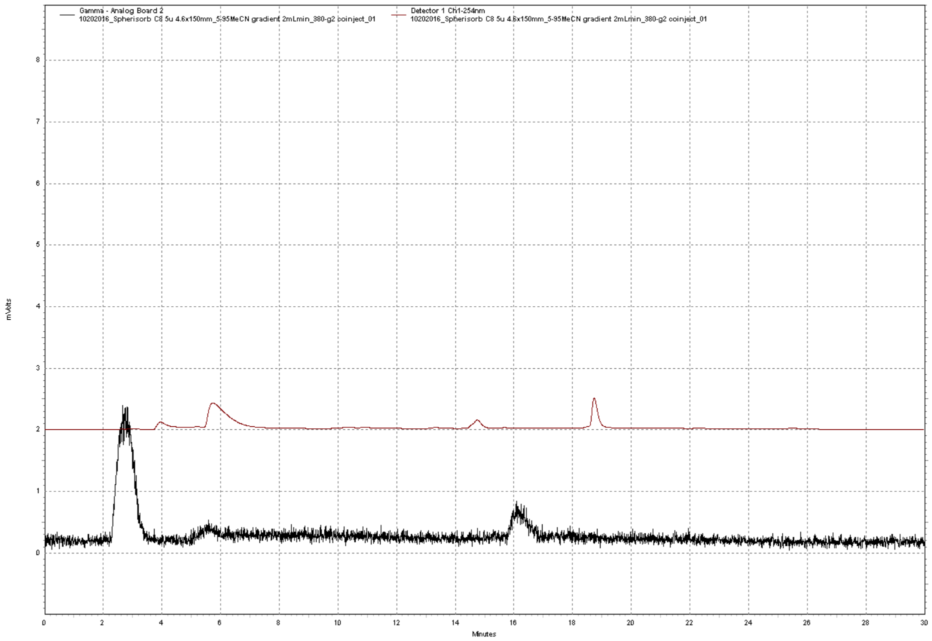

Figure 6.5C: 4-[^{18}F]-2,4-difluoroaniline (**7**) RAD trace overlaid with UV trace (256 nm) spiked with 2,4-difluoroaniline reference standard, HPLC Conditions E.

6.6: 5-[^{18}F]Fluoroindole (8)

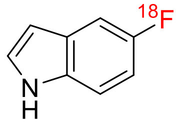

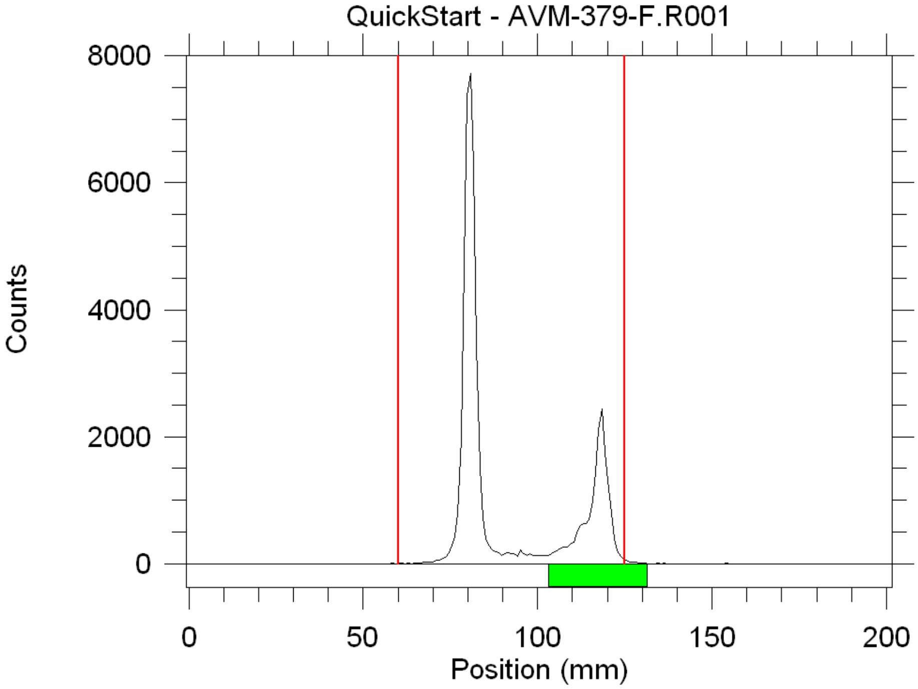

Figure 6.6A: 5-[^{18}F]fluoroindole (**8**) Radio-TLC spectrum

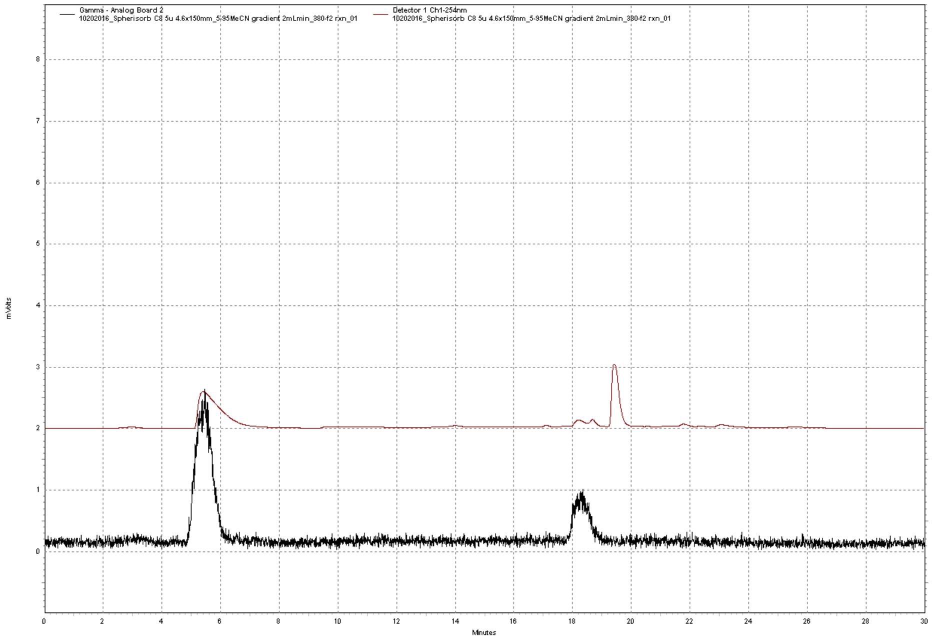

Figure 6.6B: 5-[^{18}F]fluoroindole (**8**) RAD trace overlaid with UV trace (256 nm), HPLC Conditions E

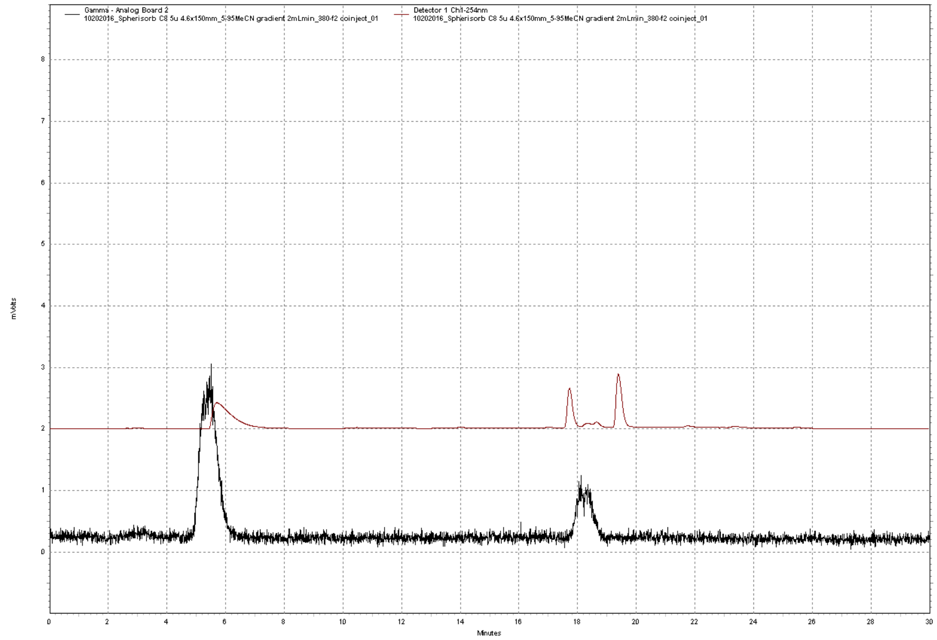

Figure 6.6C: 5-[^{18}F]fluoroindole (**8**) RAD trace overlaid with UV trace (256 nm) spiked with 5-fluoroindole reference standard, HPLC Conditions E.

7. Regression Analysis

To develop a predictive model for [^{18}F]fluoride recovery vs. pKa, regression analysis was conducted on the data collected in this experiment using GraphPad software (90% confidence level, Table 7). Regression equations were obtained with high correlation (R^2>0.9) for each preconditioning agent and serve as a crude predictor of [^{18}F]fluoride recovery when similar non-ionic eluents are use. The equations predict that bases with a conjugate acid pKa of ≥10.4 are optimal for recovering >50% [^{18}F]fluoride from ion exchange cartridges preconditioned with KOTf, whereas pKa values ≥8.9 and ≥7.7 are required when preconditioning with $NaHCO_3$ and Na_2SO_4, respectively. A similar relationship can be expressed in terms of pH instead of pKa, and the regression equations corresponding to [^{18}F]fluoride recovery versus pH (predicted from solution concentration and pKa) are also shown in Table S7. These equations are not bound by concentration and can thus predict [^{18}F]fluoride recovery from any non-ionic aqueous solution (both sets of equations approach zero at the lower conjugate pKa values and are only suitable for basic eluents. It can be envisioned that strong acid solutions that dissociate into H_3O^+ and A^- ion pairs would also be suitable eluents, likely following the same trend with pKb).

$$\%\ Fluoride\ Recovery\ =\ 100\ \div \left(1+10^{(A*(B-pKa))}\right)$$

% [^{18}F] fluoride recovery as a function of pKa (reported values)

cartridge precond. Agent	A	B	R^2
KOTf	0.508	10.38	0.976
$NaHCO_3$	0.553	8.94	0.963
Na_2SO_4	0.356	7.68	0.921

% [^{18}F] fluoride recovery as a function of pH (predicted from pKa)

cartridge precond. Agent	A	B	R^2
KOTf	1.016	11.69	0.976
$NaHCO_3$	1.105	10.97	0.963
Na_2SO_4	0.712	10.34	0.921

Table 7. Regression equation for [^{18}F]fluoride recovery vs pKa/pH of aq. base solution
